# Ionizing radiation and chemical oxidant exposure impacts on *Cryptococcus neoformans* transfer RNAs

**DOI:** 10.1371/journal.pone.0266239

**Published:** 2022-03-29

**Authors:** Melissa Kelley, Mellie June Paulines, George Yoshida, Ryan Myers, Manasses Jora, Joel P. Levoy, Balasubrahmanyam Addepalli, Joshua B. Benoit, Patrick A. Limbach

**Affiliations:** 1 Department of Chemistry, University of Cincinnati, Cincinnati, Ohio, United States of America; 2 Department of Biological Sciences, University of Cincinnati, Cincinnati, Ohio, United States of America; University of the Philippines Diliman, PHILIPPINES

## Abstract

*Cryptococcus neoformans* is a fungus that is able to survive abnormally high levels of ionizing radiation (IR). The radiolysis of water by IR generates reactive oxygen species (ROS) such as H_2_O_2_ and OH^-^. *C*. *neoformans* withstands the damage caused by IR and ROS through antioxidant production and enzyme-catalyzed breakdown of ROS. Given these particular cellular protein needs, questions arise whether transfer ribonucleic acids molecules (tRNAs) undergo unique chemical modifications to maintain their structure, stability, and/or function under such environmental conditions. Here, we investigated the effects of IR and H_2_O_2_ exposure on tRNAs in *C*. *neoformans*. We experimentally identified the modified nucleosides present in *C*. *neoformans* tRNAs and quantified changes in those modifications upon exposure to oxidative conditions. To better understand these modified nucleoside results, we also evaluated tRNA pool composition in response to the oxidative conditions. We found that regardless of environmental conditions, tRNA modifications and transcripts were minimally affected. A rationale for the stability of the tRNA pool and its concomitant profile of modified nucleosides is proposed based on the lack of codon bias throughout the *C*. *neoformans* genome and in particular for oxidative response transcripts. Our findings suggest that *C*. *neoformans* can rapidly adapt to oxidative environments as mRNA translation/protein synthesis are minimally impacted by codon bias.

## Introduction

*Cryptococcus neoformans* is a fission yeast capable of withstanding a multitude of environmental conditions including the ionizing radiation (IR) present in Chernobyl, Ukraine [1]. Despite constant exposure to IR, *C*. *neoformans* and other fungi were found to be thriving at the nuclear accident site. Notably, this fungus survives IR doses substantially higher than the lethal dosage for humans [1, 2]. Ionizing radiation impacts biological organisms directly and indirectly [3]. The direct effect of IR is the result of γ-rays lysing biomolecules causing spontaneous gene mutations and DNA lesions [4]. The indirect effect of IR is through the radiolysis of water which generates free radicals and reactive oxygen species (ROS), such as OH^-^ and H_2_O_2_ [5]. Subsequent reactions of H_2_O_2_ with iron in the cell produces additional OH^-^ radicals through Fenton chemistry [6, 7]. While endogenous ROS is typical for cellular processes [8], an imbalance of ROS can lead to cellular damage via oxidative stress [9]. Therefore, to survive IR exposure, the cell must withstand abnormally high ROS levels and γ-ray lysis. In *C*. *neoformans* and other fungi, mechanisms for addressing excess ROS include antioxidant production, enzymatic removal, and the alternative oxidase pathway for ATP [10]. For example, the enzyme catalase reacts with H_2_O_2_ to form O_2_ and H_2_O and is upregulated in *C*. *neoformans* in response to H_2_O_2_ treatment [11]. Regardless of the antioxidant mechanism, protein production is crucial for adaptation to an oxidative environment where increasing levels of proteins become necessary to prevent and repair oxidative damage.

Transfer RNA (tRNA) is the carrier of amino acids to the translational machinery in protein synthesis. The anticodon of tRNA decodes the three-nucleotide codon on mRNA and the appropriate amino acid is added to the peptide chain. Chemical modifications to tRNA are required to stabilize structure, and anticodon modifications account for the degeneracy within the genetic code [12, 13]. Many microorganisms are known to utilize tRNA modifications in response to adverse conditions. For instance, 2’-O-methyl modifications are used to stabilize tRNA at higher temperatures [14, 15]. In *Escherichia coli*, ultra-violet (UV) radiation affects RNA modifications by oxidizing susceptible nucleosides (i.e., thiolations) which causes the formation of oxidation products in tRNA [16]. In the yeast *Saccharomyces cerevisiae*, tRNA modification abundances are altered nonrandomly by exposure to alkylating and oxidizing chemical agents including H_2_O_2_ [17, 18]. In H_2_O_2_ treatment, there is a coordinated induction of the tRNA modifications 5-methylcytidine (m^5^C), 2’-O-methylcytidine (Cm), and *N2*,*N2*-dimethylguanosine (m^2^_2_G) while the alkylating agents induced a different subset of tRNA modifications [17]. Over the conditions surveyed in *S*. *cerevisiae*, 23 tRNA modifications exhibited abundance changes–many in a stress-specific manner [19]. Altogether, modifications are regulators of translation and contribute to the overall stability of tRNA. Moreover, evidence suggests tRNA modifications are part of a strategy utilized by microorganisms to survive more extreme environmental conditions.

In this work, the impact of high ROS conditions on tRNA modifications was evaluated in *C*. *neoformans*. Utilizing liquid chromatography tandem mass spectrometry (LC-MS/MS), the tRNA modifications in *C*. *neoformans* were first characterized. Next, *C*. *neoformans* was exposed to H_2_O_2_ and IR and the abundances of tRNA modifications were measured. To ensure variations in modification levels were properly understood, changes to the tRNA pool composition in response to oxidative conditions were determined. These data revealed that *C*. *neoformans* does not require adaptation of the tRNA pool or tRNA modification levels in response to the oxidative conditions. A potential explanation for this unique finding that involves the translational requirements of *C*. *neoformans* is presented.

## Materials and methods

### Culture and growth conditions

*Cryptococcus neoformans* JEC21 serotype D (MYA-565, ATCC, Manassas, VA) was grown in autoclaved potato dextrose (PD) medium (Sigma Aldrich, St. Louis, MO) by adding 23.5 g of PD to 1 L H_2_O (pH = 5.5). For agar plates, 2% w/v of agar was added to the liquid media prior to autoclaving. A 15 μL aliquot of stock cells was quadrant streaked onto a PD 2% agar plate. Two days later, an isolated colony was selected and inoculated into 0.5 L of PD liquid medium at 30°C shaking at 180 rpm. Growth curve data were generated using an absorbance microplate reader (800TS, BioTek, Winooski, VT). Cells were harvested in mid-log phase or approximately 15 h after inoculation (**S1 Fig**) and washed with sterile 1xPBS (10xPBS stock in-house of 500 mL: 40 g NaCl, 1 g KCl, 7.2 g Na_2_HPO_4_, 1.2 g KH_2_PO_4_, pH to 7.4, autoclaved). The strains of *C*. *neoformans* and *Saccharomyces cerevisiae* referenced in this study are listed in **S1 Table**.

### Hydrogen peroxide exposure conditions and viability assays

One colony was inoculated and grown to mid-log phase in PD medium. Growth from one colony subjected to H_2_O_2_ treatment was considered to be one biological replicate. Prior to H_2_O_2_ exposure, cells were counted using a trypan blue stain (0.4% Thermo Fisher Scientific, Waltham, MA) and hemocytometer (Bright-Line, Hausser Scientific, Horsham, PA). Briefly, 10 μL of cells were diluted 1:1,000 in 1xPBS and then diluted again 1:2 with trypan blue stain. The average number of cells in the four quadrants were used to calculate the cell count. To obtain the number of cells per mL, the average cell count was multiplied by 10^4^ and then multiplied by the dilution factor.

The stock H_2_O_2_ 30% solution (8.8 M) (Sigma Aldrich) was diluted to 0.5 M H_2_O_2_ to make a working solution. Immediately, the working solution was diluted with PD medium to the desired concentrations. For the viability curve the following concentrations were used: 2 mM, 5 mM, 12 mM, 20 mM, 50 mM, 200 mM, and 250 mM H_2_O_2_. The concentrations of 2 mM, 5 mM, and 12 mM were selected based on cytotoxicity of *S*. *cerevisiae* exposed to H_2_O_2_ [17]. The same number of *C*. *neoformans* cells (10^6^ cells/mL) were centrifuged for 15 min at 12,000 x g, the media washed off with 25 mL of 1xPBS, pelleted again, and resuspended in the appropriate H_2_O_2_ mixture. The vials were incubated at 30°C shaking at 180 rpm for 1 h.

To assess viability, 100 μL of cells were collected after 55 min of H_2_O_2_ exposure from each treatment. Aliquots of cells were diluted with 1:10 with 1xPBS and then 1:2 with trypan blue dye. Then 10 μL were counted with the hemocytometer under the assumption the blue cells were non-viable and clear cells were viable. The average count of the four quadrants were used to calculate percent viability by dividing viable cell number by the total cell number and multiplying by 100. After 1 h exposure, the cells were washed with 1xPBS and frozen with liquid N_2_ until further processing. H_2_O_2_ exposures were performed on three biological replicates and significance was considered by Student’s t-test with a p-value < 0.05.

### Ionizing radiation exposure conditions and viability assays

*C*. *neoformans* was grown to mid-log phase, aliquoted into glass vials (1.5x10^8^ cells/mL), and exposed to IR using a Cobalt-60 (^60^Co) source at a rate of 132 Gy/h. Cells were irradiated in PD medium at 50 Gy, 100 Gy, 150 Gy, 200 Gy, and 300 Gy doses. The cells were washed with 1xPBS and processed for total RNA and tRNA isolation. To assess viability, an aliquot of exposed cells was serially diluted (1:1,000, 1:10,000, and 1:100,000) and 150 μL were plated on PD agar plates. Two days later, the colony forming units (CFUs) were counted and used to calculate viability with the dilution factor. A Student’s t-test on the percent viability with a p<0.05 of three replicates was considered to be significant.

### Total RNA and tRNA isolation

Total RNA was isolated from frozen cell pellets using the miRvana miRNA isolation kit (Thermo Fisher Scientific) following the manufacturer’s protocol with minor adjustments. Briefly, frozen cell pellets were resuspended in 3 mL of lysis buffer. A sequence of liquid N_2_ followed by homogenization with the mortar and pestle was repeated three times. The manufacturer’s protocol was followed until the second ethanol precipitation where the aqueous-ethanol mixture was passed through the same column and the flow thru was discarded. Next, the column was washed with the kit buffers and RNA was eluted using 100 μL of warm H_2_O. Concentration and purity were assessed using a nanodrop spectrometer (NanoPhotometer, Pearl, Implen, Westlake Village, CA) and confirmed with a 1% agarose gel.

tRNAs were purified from the aforementioned total RNA using anion exchange chromatography with an AX20 column (Macherey-Nagel, Duren, Germany). Briefly, 20 μg of total RNA was resuspended in 1 mL of 100 mM Tris acetate (Research Products International, Mt Prospect, IL) pH 6.3 and 15% v/v ethanol (Thermo Fisher Scientific) solution. The column was equilibrated by 6 mL of 200 mM NH_4_Cl (Sigma Aldrich), 100 mM Tris acetate pH 6.3, and 15% v/v ethanol solution. The total RNA sample was passed through the column three times. Small RNAs were separated out with 6 mL of 400 mM NH_4_Cl, 100 mM Tris acetate pH 6.3, and 15% v/v ethanol. The tRNA fraction was eluted in 650 mM NH_4_Cl, 100 mM Tris acetate pH 6.3, and 15% v/v ethanol solution and precipitated by adding 1.5 volume of isopropyl alcohol and incubated at -20°C overnight. The samples were centrifuged for 30 min at 12,500 x g and the supernatant was discarded. The tRNA was dried and resuspended in H_2_O.

To eliminate possible DNA contamination, tRNA was DNase-treated with DNA-free DNA Removal Kit (Invitrogen, Carlsbad, CA) by following the manufacturer’s protocol. After obtaining the concentration via nanodrop, DNA removal was confirmed by gel electrophoresis on a 1% agarose gel.

### Quantitative PCR (qPCR)

Total RNA for qPCR analysis was isolated by resuspending the frozen cells in 10 mL of Tri reagent (Sigma Aldrich). Liquid N_2_ and a mortar and pestle were used to lyse the cells and repeated five times. After the homogenate was transferred to new tubes (1 mL/tube), 300 μL of chloroform (Thermo Fisher Scientific) was added. The mixture was vortexed vigorously and incubated at room temperature for 10 min. The mixture was centrifuged for 15 min at 12,500 x g at 4°C. The aqueous layer was removed and incubated with 1 volume of 95% ethanol for 15 min at room temperature. The mixture was then passed through a NucleoSpin RNA Mini kit (Macherey-Nagal) column. From there, the manufacturer protocol was followed which included an rDNase treatment step and wash steps. Total RNA was eluted in 60 μL of RNase free H_2_O. RNA integrity and quantity were assessed via nanodrop and confirmed with a 1% agarose gel.

Total RNA (1 μg) was converted to cDNA using iScript cDNA Synthesis Kit (Bio-Rad, Hercules, CA) following the manufacturers protocol. cDNA was diluted to a final concentration of 3 ng/μL. The genes for GAPDH and CAT1 were obtained from the annotated *C*. *neoformans* genome. The gene for Trm7, a tRNA methyltransferase, was obtained by using *S*. *cerevisiae* Trm7 as a query sequence to identify the homologous gene in *C*. *neoformans*. Primers were identified using Primer-BLAST [20] using default parameters and are listed in **S2 Table**. The stock primers were diluted to 100 μM prior to use. Samples were assessed on Stratagene Mx3005 (Agilent Technologies, Santa Clara, CA) using iTaq Universal SYBR Green Supermix (Bio-Rad). Results were assessed using the ΔΔCt method and normalized to GAPDH [21].

### Transcriptome and tRNA sequencing

To generate transcriptome data, duplicate biological samples exposed to IR doses of 0 Gy, 100 Gy, and 300 Gy were submitted to the DNA Sequencing and Genotyping at Cincinnati Children’s Hospital Medical Center (CCHMC). Total RNA was prepared at a final concentration of 1μg/μL. The RNA quality and quantity were assessed by a 2100 Bioanalyzer (Agilent Technologies). Only samples with an RQN (RNA quality number) > 8 were processed for library generation. The isolation of mRNA, cDNA synthesis, ligation and PCR amplification for library generation are described in the protocol by Illumina Truseq Stranded mRNA Sample Preparation guide. Briefly, polyadenylated RNA was probed out using oligo-dT attached magnetic beads. The mRNA was fragmented, and the first strand of cDNA synthesis was made using a reverse transcriptase, the second cDNA follows, including several quality control steps, then adapters were ligated. cDNA was amplified by PCR to create the final cDNA library.

The barcoded library was sequenced using an Illumina HiSeq 2500 read 75bp (single read). The reads for mRNA were about 30M. The Bioinformatics Services of the CCHMC DNA Sequencing and Genotyping Core facility performed the data analysis with the reference genome for *C*. *neoformans* JEC21 from EnsemblFungi (http://fungi.ensembl.org) GCA 000091045.1.3. The FASTQ files were obtained from the core facility of CCHMC. Quality control steps were evaluated for overall quality of the reads of the FASTQ files. Upon passing basic quality matrices, the reads were trimmed to remove adapters and low-quality reads using Trimmomatic. The trimmed reads were then mapped to the reference genome using Tophat (Bowtie in the background); this step created a summary of alignment and output (BAM) files for downstream processing. Then the transcript/gene quantification was determined using Cufflinks, where the output is abundance in FPKM (fragment per kilobase of exon per million reads). The quantified sample matrix was then used to determine differential expression between experimental groups (control and treated samples). The R package EBSeq was used to perform the differential gene expression analysis between the biological replicates. The significant differentially expressed genes were obtained by using a fold change cutoff of 1.5 and adjusted p-value cutoff of < 0.05. Downstream functional annotation of a gene of interest was determined using gene ontology and pathway analysis along with the Fungifun2 tool.

For tRNA abundances, total tRNA were isolated as previously described, dried, and resuspended to a final concentration of 1 μg/μL. RNA integrity was evaluated as previously described. The RapidSeq High Yield Small RNA Prep Kit (Biochain, Newark, CA) protocol was used with an added denaturing step. tRNA was denatured by incubating at 95°C for 5 min followed by immediately cooling on ice for 10 min. Size selection between 125 nt and 310 nt was utilized to obtain tRNA and adapter sequences. An Illumina HiSeq 2500 Sequencing System (Illumina, San Diego, CA) was used and generated 75bp sequence reads (total of 20 million reads).

Raw data was obtained from the CCHMC DNA Sequencing and Genotyping Core and the reads were mapped to mature tRNA sequences from tRNAscan-SE gene predictions for *C*. *neoformans* JEC21 [22, 23]. The raw data from the CCHMC DNA Sequencing and Genotyping Core for three replicates exposed to H_2_O_2_ and two replicates exposed to IR were imported into CLC Genomics Workbench 20.0 (https://digitalinsights.qiagen.com) for processing and analysis. Low quality and short reads were removed by filtering. Mapping and processing of reads were performed using previously described methods [24–26], where the match had to be 100% between each read and the tRNA sequence. tRNA abundance levels were determined by Reads Per Kilobase of transcript per Million mapped reads (RPKM) and an EDGE test was used to compare treated expression values to the control with significance determined using a fold change cutoff of 2 and a p-value < 0.05. Mature tRNA sequences were derived from the Heatmap plots for visualization of expression using the pheatmap package in R.

### Nucleoside analysis and relative quantification

Three replicates of tRNA were digested into nucleosides using previously reported conditions [27]. Briefly, 2 μg of tRNA were incubated at 95°C for 10 min and immediately cooled for 10 min at -20°C. Next, tRNA was incubated with 1/10 volume of 0.1 M ammonium acetate (Sigma Aldrich) pH 5 and nuclease P1 (2 U/μg tRNA, Sigma-Aldrich) at 45°C. After 2 h, 1/10 volume of 1 M ammonium bicarbonate (Sigma Aldrich) pH 8 was added. Snake venom phosphodiesterase (1.2x10^-4^ U/μg tRNA) was also added to catalyze formation of individual nucleotides. Phosphates were removed by addition of bacterial alkaline phosphatase (0.1 U/μg tRNA), and the mixture was incubated for an additional 2 h at 37°C and then vacuum dried.

Nucleosides were resuspended in mobile phase A and separated via reversed-phase liquid chromatography (RP-LC) using a high-strength silica column (Acquity UPLC HSS T3, 1.8 μm, 1.0 mm× 50 mm, Waters) with an ultra-high-performance liquid chromatography (UHPLC) system (Vanquish Flex Quaternary). The mobile phase A composition was 5.3 mM ammonium acetate in water, pH 4.5 and mobile phase B composed of a mixture of acetonitrile/water (40:60) with 5.3 mM ammonium acetate. The following gradient conditions were used: 0% B (from 0 to 7.6 min), 2% B at 15.7 min, 3% B at 19.2 min, 5% B at 25.7 min, 25% B at 29.5 min, 50% B at 32.3 min, 75% B at 36.4 min (hold for 0.2 min), 99% B at 39.6 min (hold for 7.2 min), then returning to 0% B at 46.9 min. The flow rate was 100 μL min^-1^ and the column temperature was set at 30°C.

After chromatographic separation, an Orbitrap Fusion Lumos Tribrid mass spectrometer with an H-ESI source was used for MS data acquisition as previously reported [28]. Briefly, the analyses were carried out in positive polarity. The settings to obtain the full scan data used a resolution of 120,000, a mass range of 220–900 *m/z*, automatic gain control 7.5 x 10^4^, and injection time of 100 ms. MS/MS fragmentation was carried out with the following collision energy setting for CID 42% and the setting for HCD 80 arbitrary units. Other instrumental settings consisted of the following: quadrupole isolation of 1.5 *m/z*; ion funnel radiofrequency level of 35%; sheath gas, auxiliary gas, and sweep gas of 30, 10, and 0 arbitrary units, respectively; ion transfer tube temperature of 289°C; vaporizer temperature of 92°C; and spray voltage of 3.5 kV.

Data processing was performed using Qual Browser in Xcalibur 3.0 and three characteristics were used to identify a given nucleoside: retention time (min), molecular ion *m/z* ([MH^+^]), and fragment ion *m/z* ([BH_2_^+^]). Extracted ion chromatograms (XIC) were generated by filtering for [MH^+^]to be within 5 ppm of error for the Orbitrap Fusion Lumos Tribrid mass spectrometer. To determine relative abundance, XIC peak areas for nucleosides were integrated and normalized with the peak area for the internal standard 5-bromo-2’- deoxycytidine (5-Br-2’-dC). Statistical analyses were performed on the fold change of normalized peak areas in three replicates. Comparison of untreated to treated groups indicated significance by a fold change cutoff of 2 and Student’s t-test p-value < 0.05.

### Relative synonymous codon usage (RSCU) calculations

To assess codon usage bias (CUB), genes upregulated in response to oxidative stress induced by IR and H_2_O_2_ were identified from previous reports that used the *C*. *neoformans* H99 strain [29, 30]. Relative synonymous codon usage (RSCU) was calculated for the homologous genes in the *C*. *neoformans* JEC21 strain. The same approach was used for *S*. *cerevisiae* genes upregulated in oxidative stress [31]. RSCU was calculated using codon frequencies from the Codon Usage Database [32] and the codon frequency of the organism’s whole genome was used as a reference [33]. The CodonCount Program from the Codon Usage Database determined codon frequencies of individual genes. From there, codon frequencies were used to calculate RSCU as previously described [33].

The lists of oxidative stress genes upregulated in *C*. *neoformans* and *S*. *cerevisiae* can be found in **S3 and S4 Tables**, respectively. The eight most abundant proteins were assumed to be the eight most abundant transcripts and representative of highly expressed gene CUB in *C*. *neoformans* and *S*. *cerevisiae* [34]. The most abundant proteins for *C*. *neoformans* and *S*. *cerevisiae* are listed in **S5 and S6 Tables**, respectively. To determine if codon usage was differing between the abundant and oxidative response genes, a ΔRSCU was calculated by subtracting the RSCU of the gene of interest from the RSCU of the reference (whole genome). Therefore, a positive ΔRSCU indicates the codon is enriched in the gene of interest while a negative ΔRSCU would suggest the codon usage is lower in the gene of interest than the whole genome. A Student’s t-test p-value < 0.05 was used to determine if the codon usage for a codon differed between gene groups.

Abundant genes in *S*. *cerevisiae* demonstrated enrichment in the codon UUG. To test if the same genes in *C*. *neoformans* demonstrate bias, homologous genes were identified by a BLAST search (blastn). The sequence of *S*. *cerevisiae* genes was the query sequence and only homologues with an Expect value < 1E^-30^ were accepted to be homologous. The list of the homologues identified in *C*. *neoformans* can be found in **S7 Table**.

## Results

### Viability in oxidative conditions generated by H_2_O_2_ and IR

**Fig 1:**
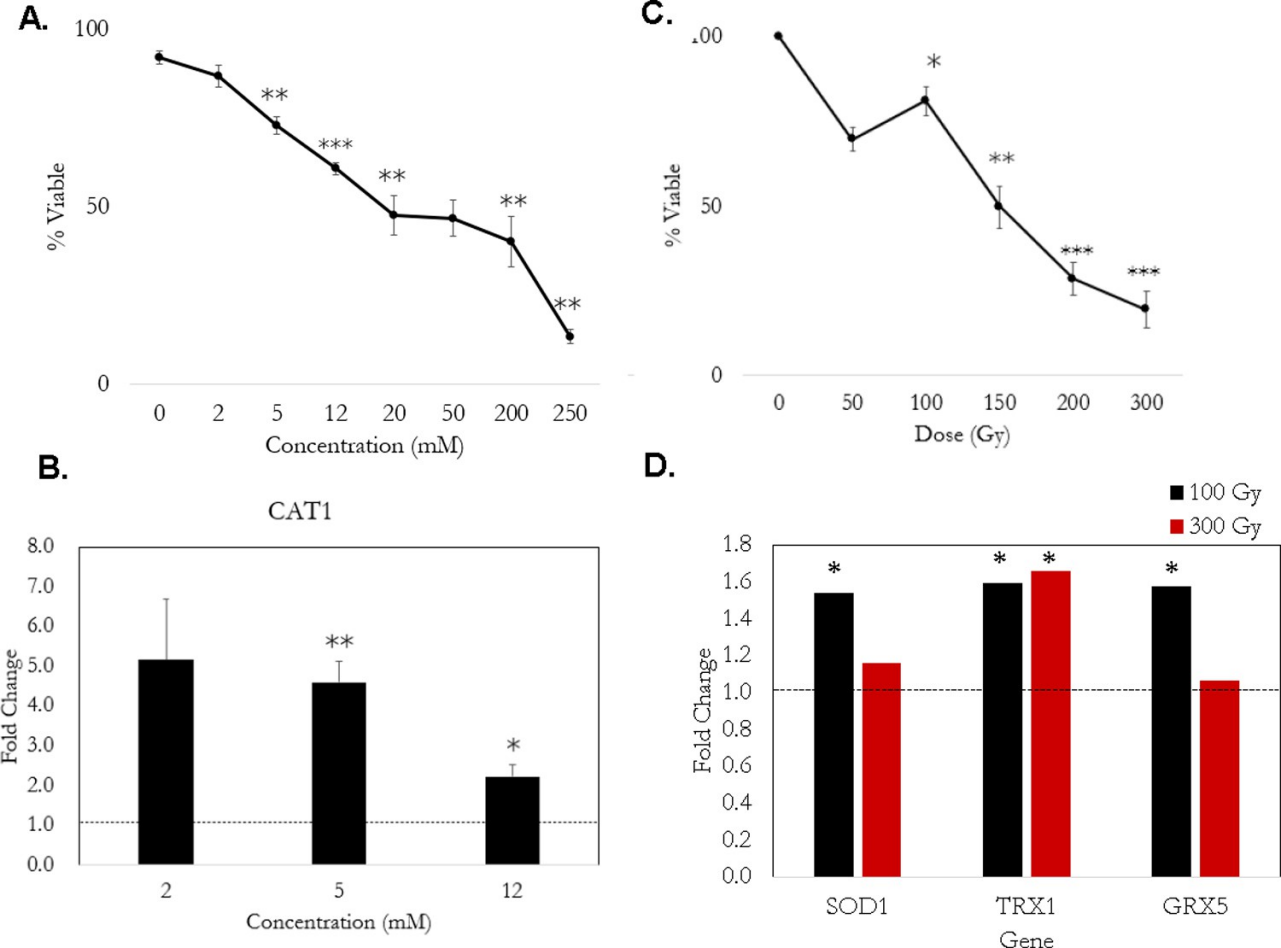
The IR and H_2_O_2_ conditions tested affect viability and gene expression of oxidative response transcripts. A. The percent viability curve in *C*. *neoformans* exposed to increasing concentrations of H_2_O_2_. Exposure was for 1 hour and viability was assessed using a trypan blue exclusion assay. Three biological replicates were considered for each dose and the error bars are the standard error of the mean (SEM). Significance was determined using a Student’s t-test and a p-value < 0.05. **B.** The fold change of catalase 1 (CAT1) gene expression in response to 2 mM, 5 mM, and 12 mM H_2_O_2_ for three biological replicates. Significance was determined by Student’s t-test with a p-value < 0.05. **C.** The average percent viability of *C*. *neoformans* exposed to increasing doses of IR via ^60^Co exposure for three biological replicates. Viability was assessed via colony forming units (CFUs) and error bars are SEM. Significance was determined using a Student’s t-test with a p-value < 0.05. **D.** Fold change of enzymes involved in IR response as determined by transcriptomics for two replicates. The significant differentially expressed genes versus no exposure controls were determined using EBseq and a fold change cutoff of 1.5 and p-value < 0.05.

To begin, the viability of *C*. *neoformans* JEC21 under oxidative conditions was assessed by exposure to concentrations of 2 mM, 5 mM, 12 mM, 20 mM, 50 mM, 200 mM, and 250 mM H_2_O_2_ for 1 h. The viability was calculated using a trypan blue exclusion assay and the percent viability decreased in a dose-dependent manner when exposed to H_2_O_2_ (**Fig 1A**). Exposure to 2 mM H_2_O_2_ did not significantly impact viability but nearly all other doses caused decreased viability. In comparison, previously published results showed *S*. *cerevisiae* exhibiting 80%, 50%, and 20% viability after 1 hour of exposure to 2 mM, 5 mM, and 12 mM, respectively [17]. Furthermore, these concentrations of H_2_O_2_ caused remodeling of tRNA modifications in *S*. *cerevisiae* [35]. In contrast, *C*. *neoformans* had 85%, 73%, and 60% viability at the same concentrations and exposure period.

Despite the relatively minimal impact of H_2_O_2_ on viability, the next step was ensuring an oxidative stress response was being triggered by the conditions tested. To assess this, the expression of enzymes known to be upregulated by H_2_O_2_ in *C*. *neoformans* was evaluated [29]. There are four catalase genes in *C*. *neoformans* and all are upregulated in response to H_2_O_2_ [11]. Catalase is an enzyme that degrades H_2_O_2_ to produce O_2_ and H_2_O and is therefore relevant to the H_2_O_2_ response [36]. To test if catalase expression was affected by the H_2_O_2_ concentrations, qPCR was utilized to assess relative gene expression of the catalase gene, CAT1. The relative gene expression of CAT1 at exposure levels of 2 mM, 5mM, and 12mM H_2_O_2_ are shown in **Fig 1B**. The 5 mM and 12 mM concentrations upregulated the expression of CAT1 in a statistically significant manner when compared to control levels. CAT1 increased 4-fold at 5 mM and 2.5-fold at 12 mM exposure over expression levels found with no H_2_O_2_ exposure for *C*. *neoformans*. However, induction of CAT1 expression with 2 mM H_2_O_2_ is highly variable with no statistical significance which coincided with the negligible impact on viability.

To determine the impact of IR on *C*. *neoformans* viability, cells were exposed to increasing concentrations of IR using gamma radiation emitted from a ^60^Co source. Post-IR exposure viability was evaluated by changes in CFUs (**Fig 1C**). Exposure to IR resulted in 80%, 29%, and 19% viability at doses of 100 Gy, 200 Gy, and 300 Gy, respectively. Thus, a more pronounced loss of viability is observed at doses above 100 Gy. These results are consistent with previous data on IR exposure in non-pigmented *C*. *neoformans* [37].

To test if the oxidative stress response was triggered by the IR conditions, *C*. *neoformans* was exposed to 100 Gy and 300 Gy doses and changes in gene expression were evaluated using RNA-seq. While CAT1 expression was not impacted by IR conditions, other oxidative response genes such as superoxide dismutase (SOD1), a thioredoxin gene (TRX1), and a glutathione peroxidase gene (GPX5) were upregulated as compared to unexposed controls (**Fig 1D**). SOD1 has a protective role in *S*. *cerevisiae* and also controls endogenous ROS during IR exposure [38]. Similarly, glutathione peroxidases reduce hydroperoxides to water using glutathione as the reductant [39]. Therefore, IR induces gene expression for enzymes that have antioxidant functions suggesting the oxidative stress response is triggered at these conditions.

### Census of tRNA modifications in *C*. *neoformans*

After confirming *C*. *neoformans* generates an oxidative stress response upon exposure to both H_2_O_2_ and IR, we next sought to examine the impact of oxidative conditions on tRNA modifications. Because the total census of tRNA modifications for this organism was previously unknown, we first acquired data on tRNA modifications prior to any exposure to oxidative conditions. *C*. *neoformans* was grown in liquid PD medium to mid-log phase, RNA was isolated, and then tRNA was digested into nucleosides as described. Using LC-MS/MS, the modified nucleosides in *C*. *neoformans* were identified using retention time, the molecular ion [MH^+^], and the nucleobase fragment ion [BH_2_^+^] (**Table 1**). Dihydrouridine (D) and pseudouridine (Ψ) are two modifications that are essential to tRNA structure and are present in *C*. *neoformans* tRNA. The D-loop of tRNA is named for the D modifications located in this arm. Likewise, the TΨC sequence is characteristic of the TΨC-loop but Ψ has been mapped to other positions in tRNA as well [40]. Inosine (I) is present in eukaryotic RNA and is commonly mapped to the wobble position of anticodons with A at position 34 [41]. At the wobble position, I can base pair with A, C, or U and therefore expands the decoding capacity through anticodon interactions with the codon [42].

**Table 1:** The modified RNA nucleosides detected in *C*. *neoformans* tRNAs.

Name, Symbol	MH^+^	BH_2_^+^	RT (min)
Pseudouridine, Ψ	245.0773	209	1.5
Dihydrouridine, D	247.0929	115	1.5
3-methylcytidine, m^3^C	258.1089	126	4.1
5-methylcytidine, m^5^C	258.1089	126	5.2
2’-O-methylcytidine, Cm	258.1089	112	6.8
2’-O-methyluridine, Um	259.0929	113	13.7
3-methyluridine, m^3^U	259.0929	127	13.7
5-methyluridine, m^5^U	259.0929	127	8.8
Inosine, I	269.0885	137	8.2
5-aminomethyluridine, nm^5^U	274.10389	142	2.6
2’-O-methyladenosine, Am	282.1202	136	29.7
*N6*-methyladenosine, m^6^A	282.1202	150	31.3
1-methyladenosine, m^1^A	282.1202	150	5.5
1-methylinosine, m^1^I	283.1042	151	19.0
*N4*-acetylcytidine, ac^4^C	286.1038	154	21.0
*N6*,*N6*-dimethyladenosine, m^6^_2_A	296.1358	164	33.4
*N6*-hydroxymethyladenosine, hm^6^A	298.1151	166	28.7
2-methylguanosine, m^2^G	298.1151	166	21.6
2’-O-methylguanosine, Gm	298.1151	152	18.6
1-methylguanosine, m^1^G	298.1151	166	19.5
7-methylguanosine, m^7^G	298.1151	166	7.4
5-carbamoylmethyluridine, ncm^5^U	302.0987	170	2.9
*N2*, *N2*-dimethylguanosine, m^2^_2_G	312.1307	180	29.5
5-methoxycarbonylmethyluridine, mcm^5^U	317.0984	185	19.6
*N2*,*N2*,7-dimethylguanosine, m^2,2,7^G	328.162	196	19.0
5-methoxycarbonylmethyl-2-thiouridine, mcm^5^s^2^U	333.0756	201	30.3
*N6*-isopentenyladenosine, i^6^A	336.1671	204	36.5
*N6*-(cis-hydroxyisopentenyl)adenosine, io^6^A	352.162	220	33.7
2-methylthio-N6-isopentenyladenosine, ms^2^i^6^A	382.15487	250	40.7
*N6*-threonylcarbamoyladenosine, t^6^A	413.142	281	30.8
8-oxoguanosine, 8-oxoG	300.0943	168	10.7

Census of tRNA modifications detected via LC-MS/MS in *C*. *neoformans* grown in PD medium to mid-log phase. Three biological replicates were used to ensure the RNA modification list was consistent. The chemical name and short name of the modification are listed with the precursor ion *m/z* [MH^+^], fragment ion *m/z* [BH_2_^+^], and retention time (RT) in minutes.

Methylations are also commonly found in tRNA and there are numerous methylations detected in *C*. *neoformans* tRNA [43]. For example, 2’-O-ribose methylations are found across all domains of life and consist of 2’-O-methylcytidine (Cm), 2’-O-methyluridine (Um), 2’-O-methyladenosine (Am), and 2’- O-methylguanosine (Gm) [13, 44]. The 2’-O-ribose methylations of the canonicals were present and there are many methylated base modifications as well in *C*. *neoformans* tRNA The pyrimidine methylations were 3-methylcytidine (m^3^C), 5-methylcytidine (m^5^C), 3-methyluridine (m^3^U), and 5-methyluridine (m^5^U). The purine base methylations characterized consisted of *N6*-methyladenosine (m^6^A), 1-methyladenosine (m^1^A), 1-methylinosine (m^1^I), *N2*- methylguanosine (m^2^G), 1-methylguanosine (m^1^G), and 7-methylguanosine (m^7^G). One modification contained two methyl group additions, *N2*,*N2*-dimethylguanosine (m^2^_2_G). The modification m^2^_2_G is a structural modification located between the D-loop and anticodon loop of many tRNAs [28].

The remaining tRNA modifications are typically found in the anticodon stem loop (ASL). Position 34 or the wobble position of the anticodon is commonly modified to alter decoding strategies [12, 45]. The position 34 modifications detected in *C*. *neoformans* are inosine (I), *N4*-acetylcytidine (ac^4^C), 5-aminomethyluridine (nm^5^U), 5-carbamoylmethyluridine (ncm^5^U), 5-methoxycarbonylmethyluridine (mcm^5^U), and 5-methoxycarbonylmethyl-2-thiouridine (mcm^5^s^2^U). Some methylations are also reported at the wobble position such as Gm, Um, Cm, and m^5^C. However, oligonucleotide mapping would be necessary to distinguish position 34 methylations from those found elsewhere throughout the tRNA. Position 37 is also located in the ASL and serves many functions in regulating translation. The *C*. *neoformans* position 37 modifications identified were *N6*-isopentenyladenosine (i^6^A), *N6*-(cis-hydroxyisopentenyl)adenosine (io^6^A), 2-methylthio-*N6*-isopentenyladenosine (ms^2^i^6^A), and *N6*-threonylcarbamoyladenosine (t^6^A). Overall, the modification census in *C*. *neoformans* mostly aligns with those reported in other fungi [46–49].

In addition to the expected tRNA modifications, several methylated nucleosides were detected that are indicative of other RNAs/RNA fragments being present in the sample. For example, m^6^_2_A has only been reported in rRNA [47]. Its presence in this data suggests that there were rRNA degradation products coeluting with the tRNA fractions during anion exchange chromatography. Additionally, the modification *N2*,*N2*,7-trimethylguanosine (m^2,2,7^G) suggests there may be small nucleolar RNA (snoRNA) contamination as well since m^2,2,7^G is a modification that ensures snoRNA stability [50]. Another modification detected in the tRNA sample was N6-hydroxymethyladenosine (hm^6^A) which has been reported in mRNA previously [51, 52]. However, these modifications were detected at lower abundances than most other modifications, suggesting minimal contamination of the tRNA pool. Prior to LC-MS/MS, tRNA samples were also visualized on a 1% agarose gel and did not have rRNA bands which further implies any contaminants were minimal.

### tRNA modification abundances in response to H_2_O_2_ and IR

After confirming *C*. *neoformans* generated a response to oxidative conditions upon exposure to H_2_O_2_ and IR and obtaining the baseline census of modified nucleosides from tRNAs, we then asked whether those modifications vary when the organism was exposed to oxidative conditions. As in the viability experiments, *C*. *neoformans* was exposed to various amounts of H_2_O_2_ and increasing doses of IR. After exposure, cells were harvested, RNA and tRNA purified, and the total tRNAs were digested to nucleosides for analysis by LC-MS/MS wherein the abundances of modifications were determined by integrating XIC peaks. To determine relative abundance, the nucleoside peak areas were normalized with an internal standard (5-Br-2’-dC) present in all samples. The fold change was calculated by dividing the treated relative abundance with the control relative abundance.

**Fig 2:**
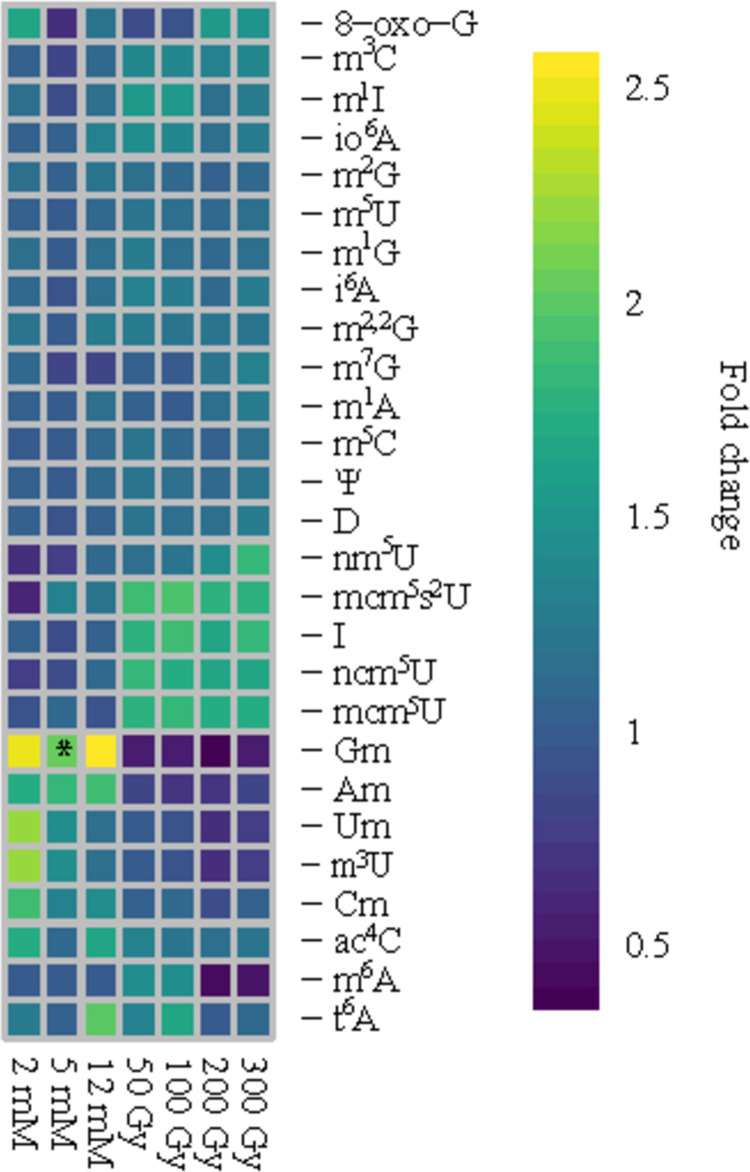
Modification abundances are largely unaffected by oxidative conditions. Heatmap of the fold change of the normalized relative abundance for the nucleosides detected in *C*. *neoformans*. The concentrations of H_2_O_2_ are 2 mM, 5 mM, and 12 mM while IR doses consisted of 50 Gy, 100 Gy, 200 Gy, and 300 Gy. The average fold change of three replicates is plotted in the heatmap. A fold change cutoff of 2 and a Student’s t-test p-value < 0.05 were considered significant.

First, we evaluated whether there were changes in the identities of modified nucleosides present due to exposure. Regardless of exposure type, no qualitative changes to tRNA modification profiles were detected (i.e., the identified modifications were identical to those in unexposed samples, **Table 1**). Next, we examined whether there were significant changes in the relative amounts of any modified nucleosides upon exposure. Notably, the majority of tRNA modifications did not vary significantly, unlike results documented in other microorganisms [19]. For instance, *S*. *cerevisiae* exposed to different environmental conditions demonstrated abundance changes for 23 tRNA modifications [17]. As shown in the heatmap of tRNA modification fold changes (**Fig 2**), most modifications did not change in abundance as compared to those from unexposed *C*. *neoformans*. While several modifications exhibited slightly higher amounts upon exposure (i.e., mcm^5^s^2^U), such changes were reproducible and dose-dependent for only a small number of modifications (Gm, Am). Overall, we did not detect dramatic changes in the identities and abundances of tRNA modifications when *C*. *neoformans* was exposed to H_2_O_2_ or IR.

### The tRNA pool composition is minimally impacted by H_2_O_2_ and IR

Next, we asked whether the pool of *C*. *neoformans* tRNA transcripts is affected by exposure to oxidative conditions. It was previously shown that *S*. *cerevisiae* remodels the tRNA pool composition to account for changes in codon demand of the oxidative response transcripts upon H_2_O_2_ exposure [53]. Likewise, an alkylating agent, hypoxia, osmotic pressure, and temperature shifts also altered the composition of the *S*. *cerevisiae* tRNA pool [53, 54]. The alterations to *S*. *cerevisiae* tRNA levels were proposed to correlate with changing codon usage bias in transcripts upregulated by the exposure conditions.

RNA-seq was utilized to measure the relative abundance of tRNAs after exposure. Transcript levels were evaluated using RPKM which is representative of relative transcript abundance and is therefore used to determine differences in tRNA expression. A fold change of 1 would indicate the exposed transcript levels were similar to the control and thereby unaltered by the treatment. A fold change value < 1 would indicate that a specific tRNA is in lower abundance in a treated versus control sample while a fold change > 1 would signify an increase in that tRNA.

**Fig 3:**
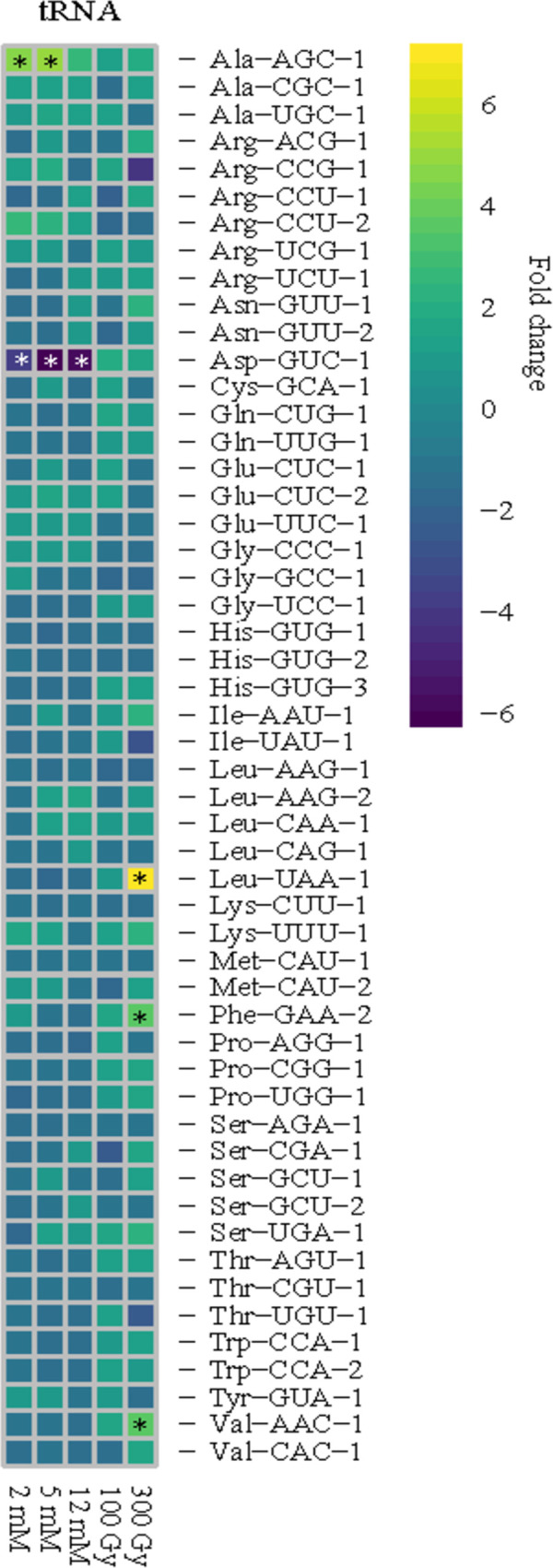
Few tRNAs have altered abundances in response to H_2_O_2_ and IR. Heatmap of the fold change for each tRNA in response to oxidative conditions. Across treatments, tRNA abundance was minimally affected in *C*. *neoformans*. Significance (*) was defined by an EDGE test with a P-value < 0.05 and a fold change cutoff of 2. Analyses were performed for three replicates in H_2_O_2_ and two replicates in IR.

First, the specific tRNAs detected by RNA-seq were identified against known tRNA gene sequences [22], and all expected tRNAs were detected except for Phe-GAA-1, which could also account for lack of yW being detected during nucleoside analysis (*cf*. **Table 1**). Further, no changes in tRNA transcript composition were noted upon exposure: the tRNAs present in the control group were also present in the H_2_O_2_ and IR-treated samples. Similar to what was noted with tRNA modifications, minimal changes in tRNA transcript abundances were found across all exposure conditions (**Fig 3**).

Altogether, the tRNA pool is unaffected by the oxidative conditions tested here with only 6% affected by IR exposure and 4% of the tRNAs affected by H_2_O_2_ (**S2 Fig**). In H_2_O_2_ exposure, only two of 54 tRNAs, Ala-AGC-1 and Asp-GUC-1, exhibited significant changes in abundance (**Fig 3**). Exposure to IR elicited a similar response with only three of 54 tRNAs affected: Leu-UAA-1, Phe-GAA-2, and Val-AAC-1 (**Fig 3**). The tRNAs affected by H_2_O_2_ and IR did not overlap suggesting that there is no coordinated change in the tRNA pool to oxidative conditions. In contrast, *S*. *cerevisiae* exposed to H_2_O_2_ resulted in 41% of the tRNAs being detected at lower abundances and 7% detected at higher abundances [53]. These changes in *S*. *cerevisiae* were observed despite the concentrations of H_2_O_2_ being lower in *S*. *cerevisiae* than *C*. *neoformans* experiments. Specifically, 0.5 mM H_2_O_2_ was enough to change the tRNA pool in *S*. *cerevisiae* [53], while here *C*. *neoformans* exhibited minimal changes even upon exposure to 5 mM H_2_O_2_. With few exceptions, the tRNA pool of *C*. *neoformans* remains relatively constant despite exposure conditions.

### Codon usage bias uniformity in abundant and oxidative response genes

The results obtained here find that *C*. *neoformans* does not use translational reprogramming to respond to oxidative conditions. Moreover, the tRNAs and tRNA modification profiles appear to be refractory to oxidative conditions–even at the most extreme conditions examined (e.g., 12 mM H_2_O_2_ or 300 Gy IR) we observed no major changes to the pool of tRNAs and their constituent modifications. It has been shown previously with other microorganisms that tRNA modifications and/or tRNA transcript levels are altered upon exposure to oxidative conditions due to codon usage changes in oxidative stress response transcripts [18, 19, 53, 55]. Thus, we next asked whether the CUB profile for *C*. *neoformans* would help explain our tRNA-related findings.

CUB is the preference of one codon over the other synonymous codons for an amino acid. All organisms have optimal codons and have different strategies for accounting for CUB [33, 56–58]. Typically, the optimal codons are used more frequently in highly expressed genes and rare codons are more likely to be in less abundant transcripts [33]. The increased usage of optimal codons in highly expressed genes is known as translational selection [57]. Many factors influence the preferred codons used and these factors include the composition of the tRNA pool, codon-anticodon interactions, gene expression, and selection pressures [59, 60]. Anticodon modifications can affect the codon preference in housekeeping conditions and unfavorable environments [61]. In microorganisms, tRNA modifications have been shown to be important in translation of codon-biased transcripts in response to stressors [18, 35, 55, 62, 63]. Ultimately, CUB is organism-specific, and natural selection favors codon usage patterns with complementing tRNA modifications to improve translational efficiency.

RSCU was calculated for each of the 61 codons in abundant transcripts and response transcripts in *S*. *cerevisiae* S288C and *C*. *neoformans* JEC21. RSCU assumes each codon is used equally for a given amino acid in the query sequence and is a standard way of measuring codon usage [33]. Higher RSCU values are found for enriched codons and the genes with the highest abundance tend to have more preferred codons than other genes [33]. Therefore, ribosomal protein genes or the whole genome codon usages are often used as references for the expected codon usage within an organism [33]. RSCU values also determine if a codon is preferred or unpreferred in a transcript and changes to codon preference via transcript levels typically affect the demand on tRNA modification and tRNA pool composition [53, 61, 64, 65]. While more elaborate statistical methods exist for codon usage analyses, RSCU values give an initial picture of the extent of codon bias and potential tRNA demand. To begin, RSCU for transcripts of the eight most abundant proteins in *S*. *cerevisiae* were calculated [33, 54]. The most abundant proteins were assumed to be the most abundant transcripts and a reference for highly expressed transcripts. The RSCU of eight genes known to be upregulated by H_2_O_2_ were also calculated [31, 66]. The difference between the RSCU values of the whole genome and each query transcript were calculated to generate a ΔRSCU. Thus, a ΔRSCU = 0 would indicate the codon is used similarly in the query gene as it is in the whole genome.

The ΔRSCU values for the most abundant genes, eight H_2_O_2_-response genes, and eight IR-response genes in *C*. *neoformans* JEC21 indicate there are not clear differences in codon usage (**S3 Fig**). The ΔRSCU values for H_2_O_2_- and IR-response transcripts indicate that the majority of codons exhibit similar usage in the query transcripts as the whole genome with the ΔRSCU close to 0. Only five of the 61 codons (e.g., CUA, GUA, GUG, ACC, and GGG) were used differently between abundant and oxidative response transcripts in *C*. *neoformans* (**S3 Fig**). In IR-induced transcripts, 13 codons have different usage than the most abundant genes (**S4 Fig**).

The codon CUA is decoded by Leu-UAG tRNAs; however there is not a gene for this tRNA in *C*. *neoformans* [22]. Therefore, CUA is likely decoded by Leu-AAG tRNAs with the modification I at the wobble position [67]. The codons GUA and GUG are decoded by valine tRNAs, Val-CAC and Val-AAC. The presence of I34 on Thr-AGU and Val-AAC tRNAs would be necessary to decode the codons ACC and GUA, respectively [68]. The codon GGG is decoded by Gly-CCC tRNAs. The other 56 codons were not used significantly different in the oxidative response genes and abundant genes. Overall, there is not an obvious trend in codon preference in *C*. *neoformans* regardless of gene type.

To ensure this method provides us a reasonable evaluation of the organism’s CUB, the ΔRSCU values for *S*. *cerevisiae* abundant and oxidative response transcripts were calculated in the same manner as *C*. *neoformans*. The ΔRSCU values indicate that there are many differences in codon usage between the gene types in *S*. *cerevisiae* (**S5 Fig**). More bias in codon preference is observed in the most abundant transcripts because several codons are enriched (i.e., AGA, UUG, GGU) while many others are unpreferred (i.e., UGC, UUU, ACA) compared to the genome. In *S*. *cerevisiae*, 48 out of the 61 codons were used differently between abundant genes and oxidative response genes which further demonstrates the clear bias differences. Altogether, the codon usage is different between these two transcript groups in *S*. *cerevisiae* and likely contributes to the alterations to tRNA observed in response to oxidative treatments.

**Fig 4:**
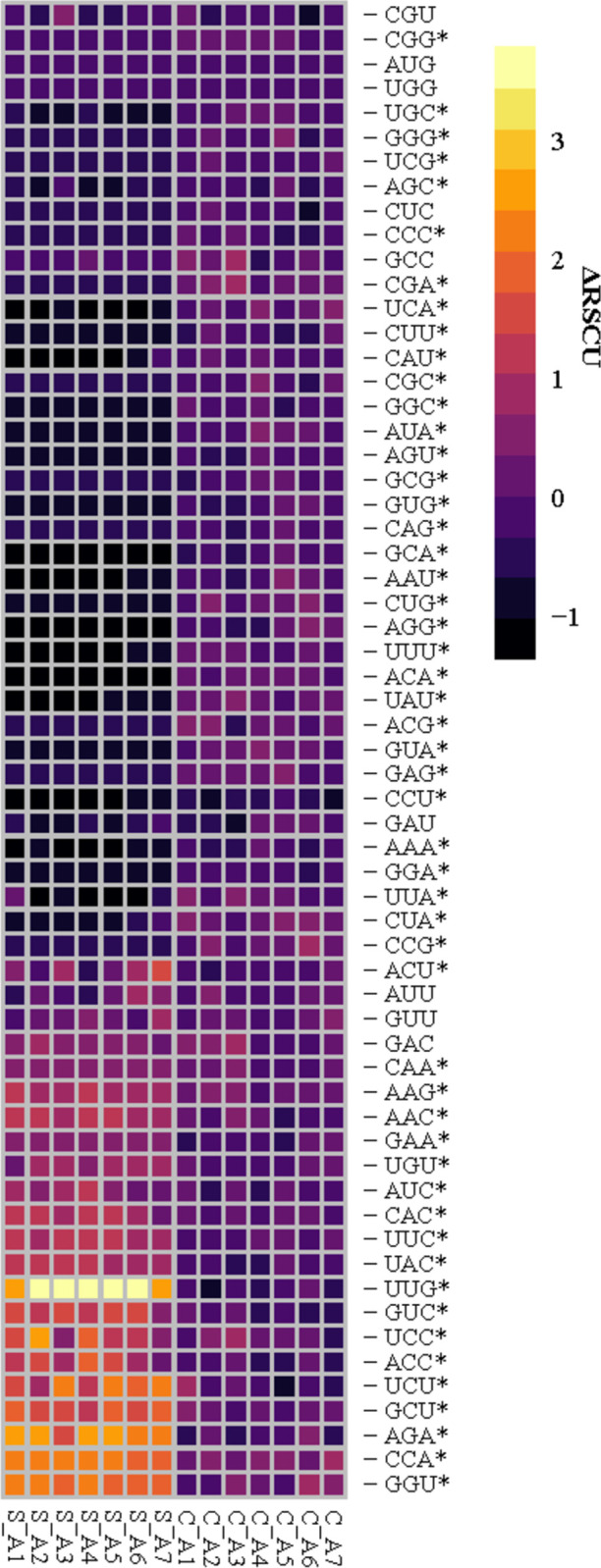
Codon usage is similar between the whole genome and oxidative response transcripts in *C*. *neoformans*. The ΔRSCU of seven UUG-rich genes in *S*. *cerevisiae* (S_A1 –S_A7) and the seven homologous genes identified in *C*. *neoformans* JEC21 (C_A1 –C_A7) (**S7 Table**). ΔRSCU values were calculated by subtracting the gene RSCU from the whole genome RSCU for each codon. Therefore, values equal to 0 indicate the codon is in similar usage as the genome. A positive ΔRSCU would indicate the codon is used more in the gene than the genome. On the contrary, a negative ΔRSCU suggests the codon is used less in the gene than the whole genome.

To support the trend of less bias in *C*. *neoformans* genes, the most abundant *S*. *cerevisiae* transcripts were used to identify the possible homologous genes in *C*. *neoformans*. The eight most abundant genes in *S*. *cerevisiae* were selected because there is obvious enrichment of the UUG codon. Since these genes demonstrate clear CUB (**S5 Fig**), the homologous genes in *C*. *neoformans* may have more bias than the other genes surveyed. The heatmap of ΔRSCU values for the UUG rich *S*. *cerevisiae* transcripts and the corresponding homologue in *C*. *neoformans* JEC21 is shown in **Fig 4**. According to ΔRSCU, *C*. *neoformans* demonstrates similar codon usage to the genome in these transcripts as well. In the homologous genes, ΔRSCU values remain close to 0 in nearly all codons in *C*. *neoformans* with only eight of the 61 codons exhibiting altered usage from the most abundant transcripts. Thus, the twenty-five genes surveyed in *C*. *neoformans* do not exhibit polarized CUB in the same manner as *S*. *cerevisiae*.

## Discussion

*C*. *neoformans* is a fission yeast that is capable of withstanding high levels of ionizing radiation and other oxidative environments. *C*. *neoformans* is able to purge media of H_2_O_2_ at concentrations lower than 8 mM in an hour [29], and we found that ~50% of cells remain viable even upon exposure to 50 mM H_2_O_2_ (**Fig 1A**). In contrast, yeast demonstrate 10% viability after 1 hour of 15 mM H_2_O_2_ exposure [17], and mouse lymphocyte cells reach 10% viability after 1 hour of exposure to 1 μM H_2_O_2_ [69]. When exposed to IR, *C*. *neoformans* was viable even at doses as high as 300 Gy (**Fig 1C**) which is consistent with previous results [37]. *S*. *cerevisiae* have approximately 10% viability when exposed to 200 Gy [70]. In contrast, humans experience acute radiation sickness at doses over 2 Gy and doses over 10 Gy are usually lethal [71]. Altogether, *C*. *neoformans* demonstrates enhanced resistance to H_2_O_2_ and IR exposure compared to other organisms.

*C*. *neoformans* responds to oxidative conditions by upregulating genes related to antioxidant production, ROS-management, and DNA repair mechanisms. For instance, in response to H_2_O_2_ and IR exposure DNA repair genes (i.e., RAD proteins) are induced and RAD54 is required for radiation resistance [29, 30]. Here, we also observed higher transcript levels of RAD proteins in response to IR (**S6 Fig**). Catalase is another enzyme that is critical in response to H_2_O_2_ in *C*. *neoformans* [11, 29], and we also observed increased levels of CAT1 in H_2_O_2_ conditions (**Fig 1B**). IR did not induce CAT1 expression, which is consistent with previous transcriptome analyses in *C*. *neoformans* exposed to IR [30]. However, other important ROS regulating enzymes such as SOD1, TRX1, and GRX5 had increased expression in IR (**Fig 1D**). Although beyond the scope of this study, further examination of the protein levels would provide additional confirmation that these enzymes are both expressed and present during the conditions tested here. Overall, the consistent upregulation of enzymes critical in ROS regulation indicates that there is a response to both oxidizing treatments in *C*. *neoformans*.

Modifications to tRNA are common and necessary for their proper function. tRNA modifications generally influence translational speed and accuracy and the absence of modifications can lead to issues in protein production [12, 61, 72]. Here we report for the first time the census of modified nucleosides from *C*. *neoformans* tRNAs (**Table 1**). *C*. *neoformans* contains many of the typical structural modifications common to microorganisms [47]. Moreover, many of the ASL modifications at position 34 and 37 are similar to those found in other microorganisms. Most of these modifications are expected as they are commonly used for decoding strategies in eukaryotes (i.e., I, mcm^5^s^2^U).

At the wobble base position [34], inosine can base pair with A, C, or U and therefore expand the decoding capacity through anticodon interactions with the codon [42]. The elongator complex is conserved and required for the formation of mcm^5^U, mcm^5^s^2^U, and ncm^5^U in yeast [73]. Therefore, these modifications were expected to be present in *C*. *neoformans* as well. For example, in eukaryotes U34 can undergo a multistep, multienzyme reaction to form mcm^5^U which can then be thiolated to form mcm^5^s^2^U [47]. This occurs in three tRNAs that contain U34: lysine (Lys) with the UUU anticodon, glutamic acid (Glu) with the UUC anticodon, and glutamine (Gln) with the anticodon UUG [74]. Loss of mcm^5^s^2^U through enzyme disruption led to drastically lowered protein levels highlighting its importance in protein synthesis [74]. As another example, nm^5^U has been detected in thermophilic microorganisms such as the anaerobic bacteria, *Thermotoga maritima*, and green sulfur bacteria, *Chlorobium tepidum* [47, 75, 76]. The modification nm^5^U is primarily reported in bacteria and the enzymatic mechanism for formation of nm^5^U is unclear in eukaryotes [47, 77]. In *C*. *neoformans*, nm^5^U is possibly used as a U34 decoding strategy rather than an oxidation project of mnm^5^U but more investigation is required [16].

When evaluating position 37 modifications, the presence of t^6^A is unsurprising since this modification is universally conserved in all domains of life and stabilizes anticodon interactions [78, 79]. As methionine tRNAs contain the anticodon CAU, they decode an ANN codon and contain t^6^A [47, 80]. Detection of i^6^A was also expected as it is commonly found in eukaryotes [47] including on many tRNAs in the yeasts *S*. *cerevisiae* and *S*. *pombe* [81]. Some tRNAs that contain i^6^A in both yeast species were tyrosine (Tyr) with the anticodon GUA [47, 81]. Serine (Ser) tRNAs with the anticodon AGA also contain i^6^A37 in yeasts [47]. In mammalian tRNAs, i^6^A can be thiolated to form ms^2^i^6^A [81]. The hypermodifications io^6^A and ms^2^i^6^A are typically reported in bacteria [47] with ms^2^i^6^A not yet being identified in other yeasts [47, 81]; however, this hypermodification was detected in *C*. *neoformans*.

We expected to detect the position 37 modification wybutosine (yW) since it is known to be on phenylalanine (Phe) tRNA in eukaryotes and other yeasts [82]. The precursor to yW is m^1^G which was detected but it is unclear if it is only present on Phe-tRNA. tRNA-Phe expression may be at a lower abundance compared to the rest of the tRNA pool causing yW to be undetectable. Overall, some of the position 37 modifications were conventional for yeasts but some were observed uniquely in *C*. *neoformans*.

Previous studies have found that tRNA modification levels are altered in response to adverse conditions in other microorganisms such as *S*. *cerevisiae* and *S*. *pombe* [19, 55, 62, 63]. In *S*. *cerevisiae*, twenty-three tRNA modification abundances are remodeled in a variety of oxidative conditions and wobble position modifications are utilized to translate mRNAs that are upregulated in oxidative conditions [18, 19, 55]. For example, *S*. *cerevisiae* exposed to H_2_O_2_ has is increased levels of m^5^C on position 34 in leucine (Leu) tRNAs with the anticodon CAA [35]. Additional studies demonstrated that m^5^C on Leu-[m^5^C]AA increased translational speed in transcripts enriched in UUG codons and loss of m^5^C via enzyme disruption led to H_2_O_2_ sensitivity likely due to inefficiency in translating UUG-rich transcripts [18, 35]. Similarly, in *S*. *pombe*, mcm^5^s^2^U on Lys-UUU tRNA enhanced the efficiency of translation for two key transcription factors in response to H_2_O_2_ [55]. Loss of mcm^5^s^2^U34 led to hypersensitivity to H_2_O_2_ because of impaired translation at AAA codons [55]. Thus, tRNA modifications affect the decoding of mRNAs and have implications on survival in environmental exposures in other microorganisms.

Despite the precedence in other organisms, H_2_O_2_ exposure does not cause significant and particular differences in *C*. *neoformans* tRNA modification levels. The minor increases in 2’-O-methylation in *C*. *neoformans* tRNAs upon exposure to oxidative conditions (**Fig 2**) may serve a structural stabilization role, but given the limited abundance changes we do not consider these increased methylations necessary for proper tRNA stability and function upon exposure [47]. The abundances of the canonical 2’-O-methylations (Am, Gm, Cm, and Um) increase in response to H_2_O_2_ yet the increase was minimal for all except Gm. The tRNA-modifying enzyme that forms Gm on the wobble position of phenylalanine (Phe) tRNA in yeast is Trm7. *S*. *cerevisiae* requires Trm7 and Gm for recovery from exposure to H_2_O_2_ [83, 84]. However, Trm7 did not exhibit higher transcript levels according to qPCR in response to H_2_O_2_ (**S7 Fig**). Likewise, in transcriptomic analyses the response to IR did not affect transcriptional levels of Trm7 either (**S7 Fig**). Ultimately, the majority of tRNA modifications are not affected by H_2_O_2_ or IR treatment in *C*. *neoformans*, which suggests tRNA modifications are likely not required to be modulated to respond to oxidative environments.

Similar to tRNA modifications, the composition of the tRNA pool remained largely constant regardless of treatment in *C*. *neoformans* (**Fig 3**). On the contrary *S*. *cerevisiae* demonstrated dramatic alterations in the tRNA pool with nearly 50% of transcripts affected in response to diauxic shift, oxidative stress, osmotic stress, and temperature shift [53]. In *S*. *cerevisiae*, the majority of the tRNAs that were impacted by H_2_O_2_ were decreased (47%) [53]. The decreases in tRNAs observed in *S*. *cerevisiae* could be in part from the rapid tRNA decay (RTD) pathway utilized to quickly degrade aberrant tRNAs [85]. The RTD pathway could be tripped by hypomodification or destabilization of the acceptor or T-stems of the mature tRNA [85, 86]. 2’-O-methylations are also found on the acceptor stem of tRNAs and Am is commonly at position 4 of His-tRNAs [87]. Only one tRNA decreased indicating there is likely minimal degradation of tRNA transcripts occurring in response to oxidative conditions in *C*. *neoformans*. Altogether, the modification and transcript differences observed between H_2_O_2_ and IR in *C*. *neoformans* implies there are different mechanisms occurring to respond to the oxidative environments.

It has been previously shown that the abundance changes to the tRNA pool in *S*. *cerevisiae* are required to meet the changes in the codon usage and tRNA demand unique to oxidative response transcripts [53]. In microorganisms, highly expressed genes have codon bias that is different from minimally expressed genes [33, 61]. In the tRNA models in other microorganisms, tRNA modifications have been shown to be important in translation of codon-biased transcripts in response to stressors [18, 35, 55, 62, 63]. Likewise, tRNA transcript levels are altered due to CUB changes in oxidative stress response transcripts [18, 19, 53, 55]. While CUB is low in the genomes of the genus *Cryptococcus*, gene expression levels contribute the most to codon usage patterns [58]. Compositional bias or the composition of codons was found to have the least impact on codon usage in *C*. *neoformans* [58]. As the most crucial determinant of codon usage in the genus *Cryptococcus* is gene expression [58], tRNA modifications and transcripts may be correlated more closely with global gene expression trends.

By comparing the DRSCU heatmaps between *C*. *neoformans* (**S3 Fig**) and *S*. *cerevisiae* (**S5 Fig**), it is immediately clear that *C*. *neoformans* does not demonstrate the same codon enrichment as *S*. *cerevisiae*. This is not necessarily surprising as every organism has its own codon usage trends [32]. There is not an obvious trend in codon preference in *C*. *neoformans*–even in the most abundant transcripts. In stark contrast to *S*. *cerevisiae*, the sixteen genes evaluated in *C*. *neoformans* have codon usage that is primarily the same as each other and the whole genome. This is also different than RSCU values in other microorganisms that suggests more highly expressed genes demonstrate more codon usage bias through overabundance of preferred codons [33, 45, 88]. Examining CUB this way implies that *C*. *neoformans* transcripts do not have different codon usage between these classes of genes or the genome.

Furthermore, *C*. *neoformans* is also a human pathogen that is deadly to immunocompromised people and codon optimization is a trait of pathogens that are able to infect a wide range of hosts [89, 90]. *C*. *neoformans* demonstrated the most codon optimization and highest number of potential hosts with infections reported in 800 different species [89]. In support of this, the transcripts assessed here in *C*. *neoformans* demonstrate similar codon usage as the whole genome (**Fig 4**) that implies uniformity of codon usage which would support the ability to rapidly adapt to new conditions. A limitation to evaluating codon usage this way is that only individual genes have been surveyed. While this was enough to observe CUB in *S*. *cerevisiae*, it is possible other genes in *C*. *neoformans* contain more extreme codon preferences. Additional multivariate statistical approaches in tandem with transcriptome data may increase confidence in CUB analyses in response to oxidative conditions.

Altogether, there is compelling evidence that tRNA modifications and pool composition are not required for *C*. *neoformans* to respond to the oxidative conditions tested here. Initially, the lack of tRNA modification abundance changes in response to H_2_O_2_ was surprising given what has been reported for other microorganisms [16, 19, 55]. Nevertheless, codon usage analyses revealed it is likely tRNA modification levels remain unaffected due to similar codon usage between the whole genome, abundant, and oxidative response transcripts. The tRNA pool composition is also minimally impacted in response to H_2_O_2_ and IR.

It is worth speculating why the results obtained here for *C*. *neoformans* seem to contrast with other microorganisms that have been studied previously. This stable tRNA profile may be due to the evolutionary history of *C*. *neoformans*, which evolved approximately 600 million years ago (mya) [91] (**S8 Fig**). This is in contrast to the younger, model yeasts *S*. *pombe* and *S*. *cerevisiae*, which evolved 440 mya and 330 mya, respectively [91]. Based on gene ortholog data, *C*. *neoformans* evolved in the Ediacaran Period (635 to 541 mya) [92]. During the Ediacaran Period, the oceans became heavily oxygenated and oxygen levels stabilized in the atmosphere and ocean which was an important prerequisite to animal evolution [93]. As an obligate aerobe, *C*. *neoformans* requires oxygen in order to grow and the shift in oxygen levels could have catalyzed the evolutionary radiation of *C*. *neoformans*. Increased oxygen could also imply higher levels of baseline ROS since the oxygen could react with metals (i.e., iron) through Fenton chemistry. Moreover, *C*. *neoformans* may be more resistant to an imbalance in ROS than in fungi that evolved more recently and under different environmental conditions as a highly oxygenated environment would require constant ROS management. Therefore, it is likely fungi that evolved in the presence of elevated oxygen levels would need to translate the transcripts of ROS-balancing enzymes like other housekeeping transcripts.

The similar CUB in oxidative response transcripts, the most abundant transcripts, and the genome in *C*. *neoformans* is consistent with a hypothesis that ROS-regulating genes were and are housekeeping genes. In regard to the more recently evolved fungi, ROS related transcripts were not housekeeping transcripts but specialized and triggered for oxidative conditions. Overall, the oxidative environment in the history of *C*. *neoformans* could have influenced CUB and gene expression of oxidative response transcripts. In turn, the evolutionary origins of *C*. *neoformans* may have primed the tRNA modifications and pool composition for translation of housekeeping and antioxidant genes and this may contribute to the observed resistance to IR and H_2_O_2_.

## Conclusion

We have shown that *C*. *neoformans* does not exhibit tRNA modification abundance alterations in response to the oxidative conditions assessed here. In other microorganisms, tRNA modifications have been linked to translation of codon-biased mRNAs in response to environmental stressors [17, 19]. Without the modifications to translate the codon-biased transcripts, cells demonstrated increased sensitivity to the stressor [35, 55]. Exposure to a chemical oxidant, H_2_O_2_, resulted in minimal changes to tRNA modification abundances in *C*. *neoformans*. The codon usage similarities observed in the genome and oxidative response transcripts suggests remodeling of tRNA modifications is likely unnecessary. These data suggest that *C*. *neoformans* lack of oxidative stress-specific and general genome CUB organization enables this organism to rapidly respond and adapt to changing environmental conditions. It would be of interest to determine whether selective pressure was responsible for *C*. *neoformans* lack of CUB, and these findings help better contextualize results found in other yeasts.

## Supporting information

S1 Fig Growth curve of *C. neoformans* in PD media. H_2_O_2_ exposure performed during mid-log phase (approximately at 15 h of growth).
(PDF)

S2 Fig Percentage of differentially expressed tRNA transcripts in *C*. *neoformans* in response to IR and H_2_O_2_ conditions. Red indicates the percent that are increased, and green represents the percent of tRNAs that were decreased. The percentage of *S*. *cerevisiae* tRNA transcripts affected by H_2_O_2_ is shown for comparison [55].
(PDF)

S3 Fig Heatmap of RSCU values for codons in the most abundant genes and genes upregulated in response to H_2_O_2_ in *C*. *neoformans*. The most abundant are labeled A1-A8 and the H_2_O_2_ -induced genes are labeled O1-O8. The list of genes can be found in S3 and S5 Tables. To determine if the codon was used differently between the two groups, a Student’s t-test was calculated and a p-value < 0.05 was considered significant. Five out of the sixty-one codons were used differently.
(PDF)

S4 Fig Heatmap of ΔRSCU values for codons in genes upregulated in response to IR in *C*. *neoformans*. The most abundant are labeled A1-A8 and the IR-induced genes are labeled IR1-IR8. The list of genes can be found in S3 and S5 Tables. To determine if the codon was used differently between the two groups, a Student’s t-test was calculated and a p-value < 0.05 was considered significant. Thirteen out of the sixty-one codons were used differently.
(PDF)

S5 Fig Heatmap of RSCU values for codons in the most abundant genes and genes upregulated in response to H_2_O_2_ in *S*. *cerevisiae*. The most abundant are labeled A1-A8 and the H_2_O_2_ -induced genes are labeled O1-O8. The list of genes can be found in S4 and S6 Tables. To determine if the codon was used differently between the two groups, a Student’s t-test was calculated and a p-value < 0.05 was considered significant. Forty-eight out of the sixty-one codons evaluated were used differently.
(PDF)

S6 Fig DNA-repair proteins RAD51 and RAD54 are upregulated in response to IR. According to RNA-seq analyses, the log fold change of transcript levels of RAD51 and RAD54 are higher in IR exposed *C*. *neoformans*.
(PDF)

S7 Fig Expression of 2’-O-methyl tRNA-modifying enzymes Trm7 in response to H_2_O_2_ and IR. Trm7 transcript levels were evaluated by qPCR for H_2_O_2_ (left) and transcriptomics for IR (right). Trm7 transcripts were not significantly affected in either oxidative condition in *C*. *neoformans*.
(PDF)

S8 Fig Approximate timeline of relevant fungi evolution. There is evidence that fungi were on land prior to the movement of plants. Fossil record evidence is abundant at 460 mya which further supports this accepted timeline. Data is from [91].
(PDF)

S1 Table Strains referenced and used in this study. Strains of *C*. *neoformans* and *S*. *cerevisiae* that are referenced in this work. The *C*. *neoformans* JEC21 strain was used to collect tRNA modification and transcriptome data reported here.
(PDF)

S2 Table Primers used for qPCR experiments in this study. The primers for GAPDH, CAT1, and Trm7 were designed using Primer-BLAST [20].
(PDF)

S3 Table List of the genes in *C*. *neoformans* induced by H_2_O_2_ exposure. Genes reported to be upregulated in response to H_2_O_2_ in *C*. *neoformans* [29]. These genes were used for codon usage analyses in S3 and S4 Figs.
(PDF)

S4 Table List of the genes in *S*. *cerevisiae* induced by H_2_O_2_ exposure. Genes in *S*. *cerevisiae* reported to be upregulated by H_2_O_2_ exposure [31]. These genes were used for codon usage analyses in S5 Fig.
(PDF)

S5 Table List of the most abundant proteins in *C*. *neoformans*. The most abundant proteins [34] were assumed to be representative of the most abundant transcripts and used for codon usage analyses in S3 and S4 Figs.
(PDF)

S6 Table List of the most abundant proteins in *S*. *cerevisiae*. The most abundant proteins [34] were assumed to be representative of the most abundant transcripts and used for codon usage analyses in S5 Fig.
(PDF)

S7 Table List of homologous genes identified in *C*. *neoformans*. Homologues of UUG-enriched genes in *S*. *cerevisiae* were identified in *C*. *neoformans* and used for codon usage analyses in Fig 4.
(PDF)
