# Supplementary figures and images for: Ionizing radiation and chemical oxidant exposure impacts on Cryptococcus neoformans transfer RNAs
Source: PLoS One. 2022 Mar 29;17(3):e0266239. doi: 10.1371/journal.pone.0266239 (PMC8963569; doi:10.1371/journal.pone.0266239)

S1 Fig

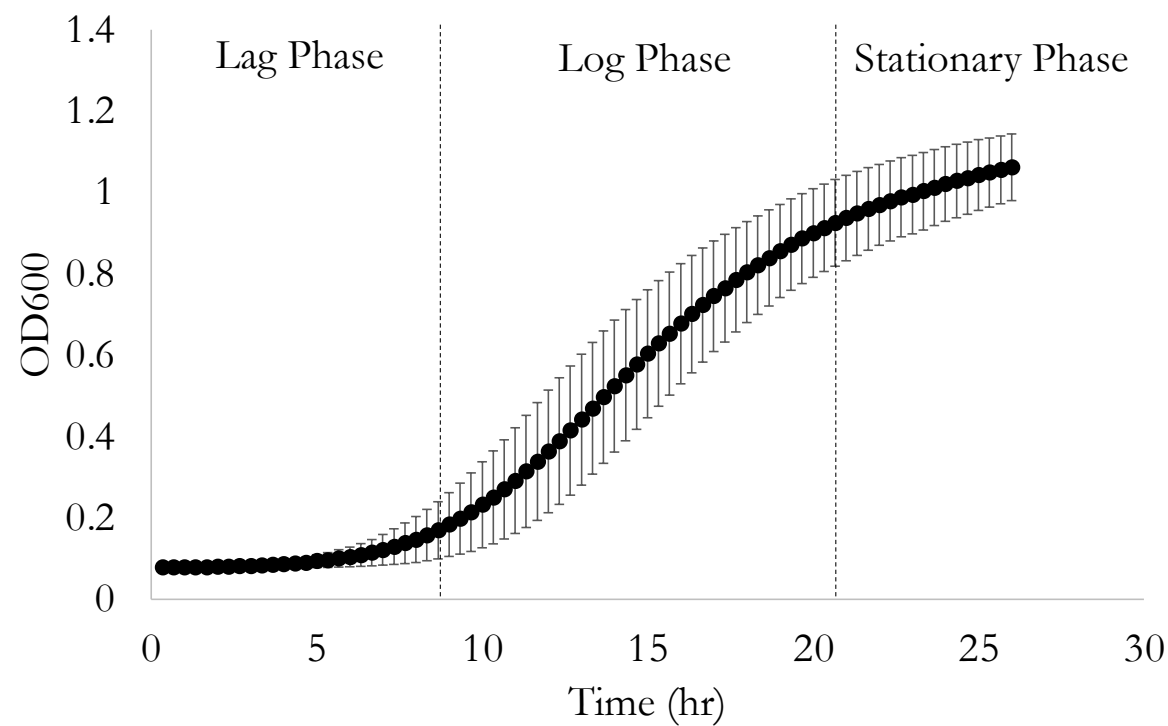

Supplement: S1 Fig — Growth curve of C. neoformans in PD media. H2O2 exposure performed during mid-log phase (approximately at 15 h of growth). (PDF) [file pone.0266239.s001.pdf]

S2 Fig

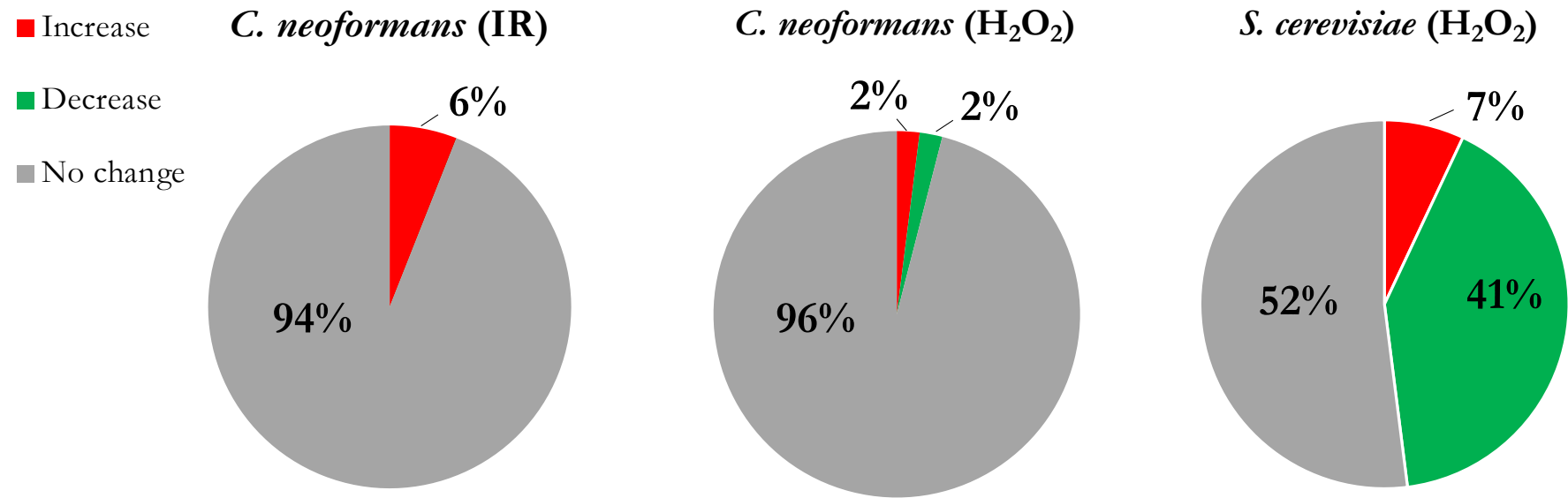

Supplement: S2 Fig — Red indicates the percent that are increased, and green represents the percent of tRNAs that were decreased. The percentage of S. cerevisiae tRNA transcripts affected by H2O2 is shown for comparison [55]. (PDF) [file pone.0266239.s002.pdf]

S3 Fig

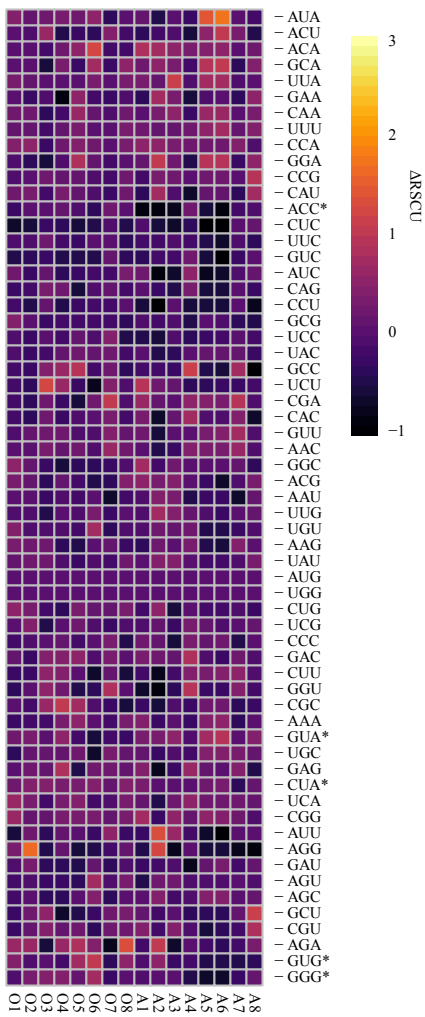

Supplement: S3 Fig — The list of genes can be found in S3 and S5 Tables. To determine if the codon was used differently between the two groups, a Student’s t-test was calculated and a p-value < 0.05 was considered significant. Five out of the sixty-one codons were used differently. (PDF) [file pone.0266239.s003.pdf]

S4 Fig

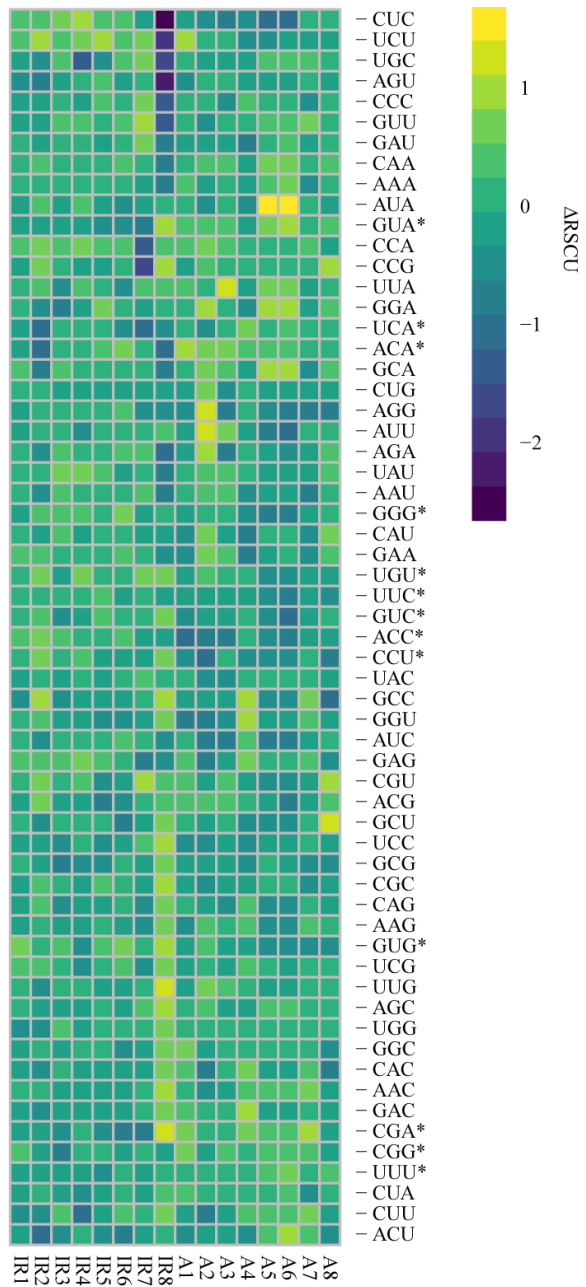

Supplement: S4 Fig — The most abundant are labeled A1-A8 and the IR-induced genes are labeled IR1-IR8. The list of genes can be found in S3 and S5 Tables. To determine if the codon was used differently between the two groups, a Student’s t-test was calculated and a p-value < 0.05 was considered significant. Thirteen out of the sixty-one codons were used differently. (PDF) [file pone.0266239.s004.pdf]

S5 Fig

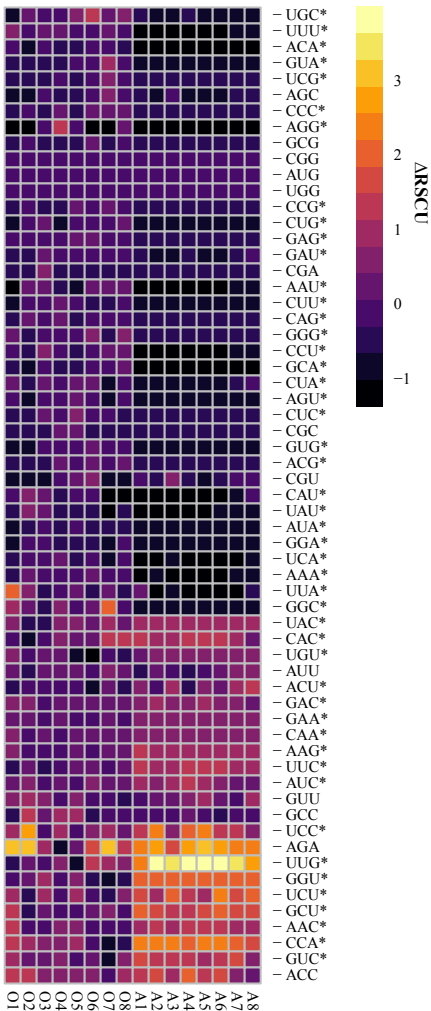

Supplement: S5 Fig — The most abundant are labeled A1-A8 and the H2O2 -induced genes are labeled O1-O8. The list of genes can be found in S4 and S6 Tables. To determine if the codon was used differently between the two groups, a Student’s t-test was calculated and a p-value < 0.05 was considered significant. Forty-eight out of the sixty-one codons evaluated were used differently. (PDF) [file pone.0266239.s005.pdf]

S6 Fig

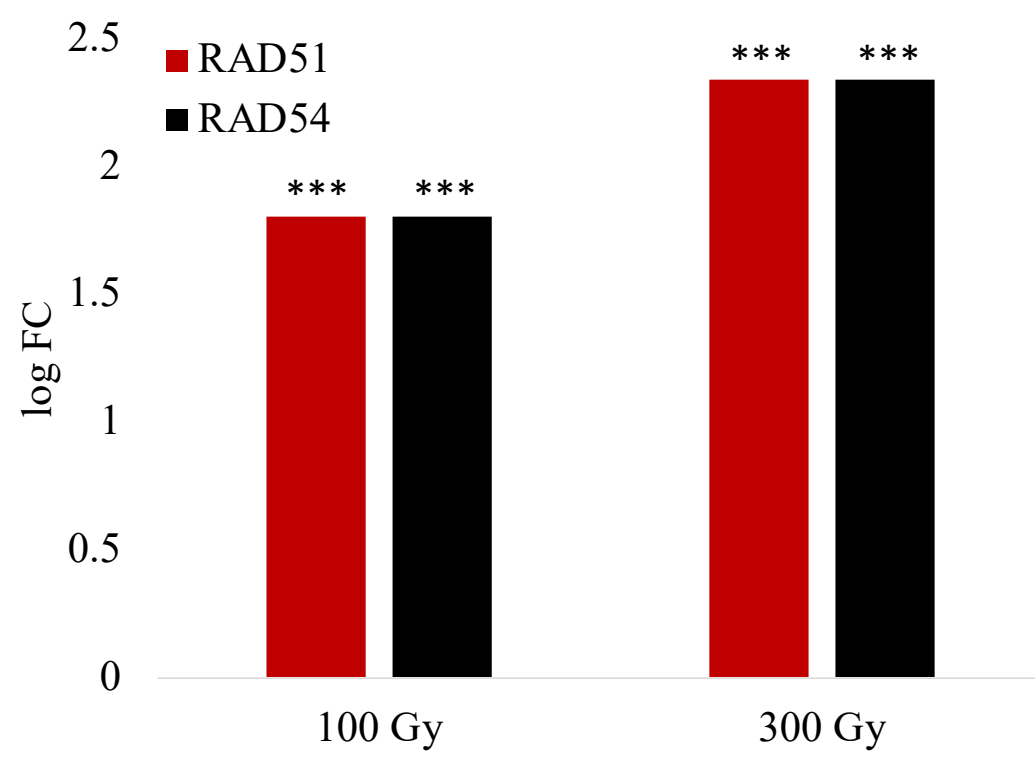

Supplement: S6 Fig — According to RNA-seq analyses, the log fold change of transcript levels of RAD51 and RAD54 are higher in IR exposed C. neoformans. (PDF) [file pone.0266239.s006.pdf]

S7 Fig

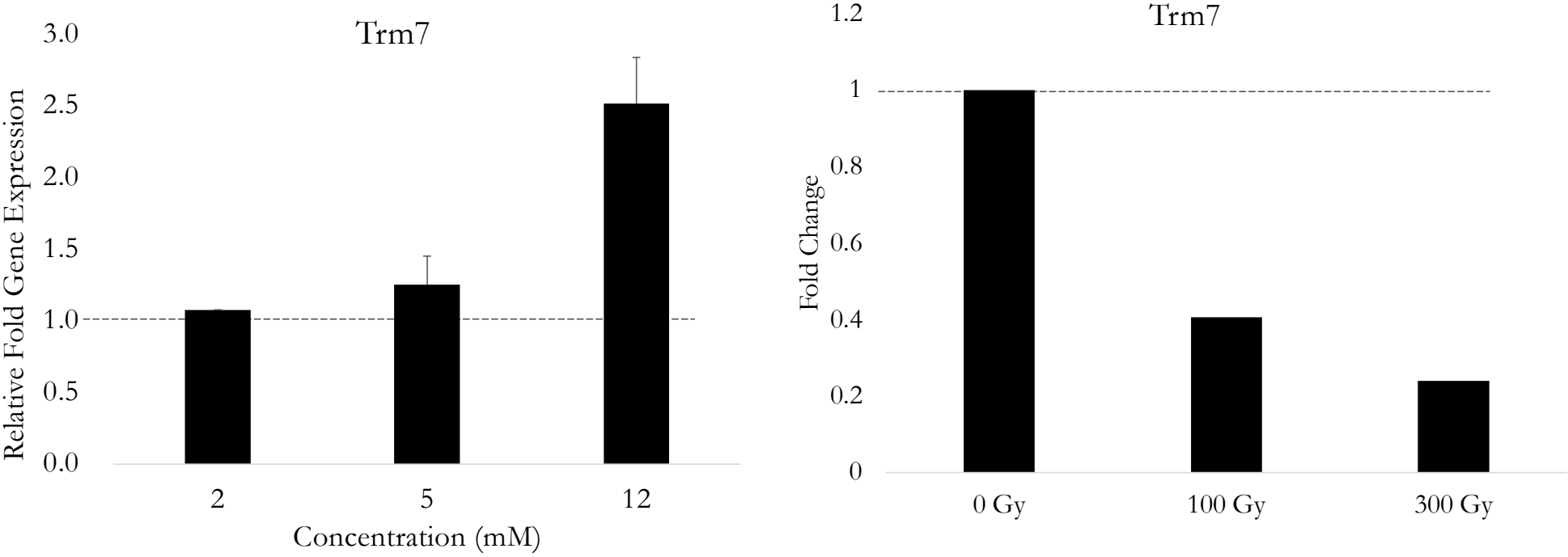

Supplement: S7 Fig — Trm7 transcript levels were evaluated by qPCR for H2O2 (left) and transcriptomics for IR (right). Trm7 transcripts were not significantly affected in either oxidative condition in C. neoformans. (PDF) [file pone.0266239.s007.pdf]

S8 Fig

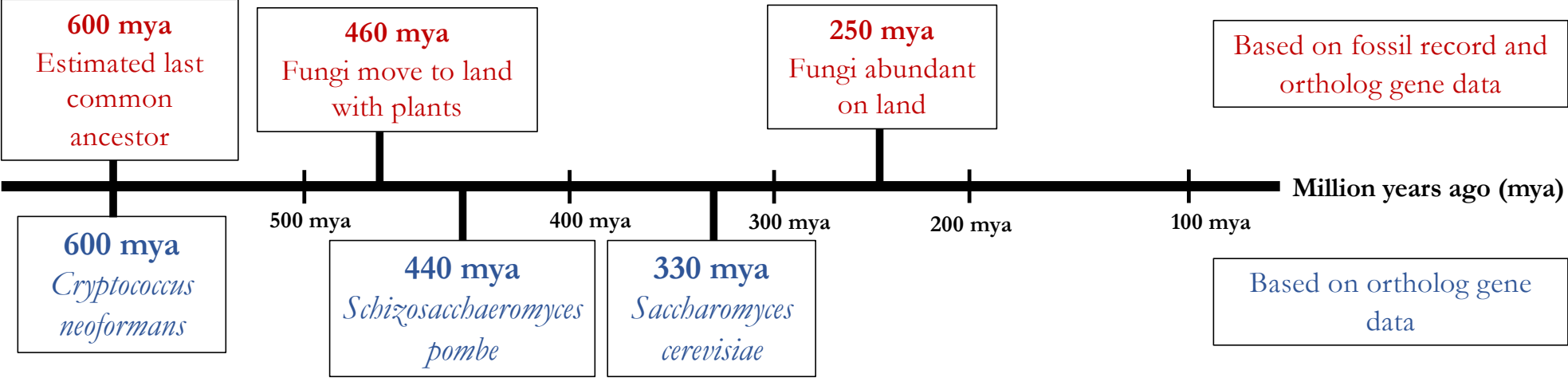

Supplement: S8 Fig — There is evidence that fungi were on land prior to the movement of plants. Fossil record evidence is abundant at 460 mya which further supports this accepted timeline. Data is from [91]. (PDF) [file pone.0266239.s008.pdf]
