# Supplementary material for: Ionizing radiation and chemical oxidant exposure impacts on Cryptococcus neoformans transfer RNAs
Source: PLoS One. 2022 Mar 29;17(3):e0266239. doi: 10.1371/journal.pone.0266239 (PMC8963569; doi:10.1371/journal.pone.0266239)
Supplement: S1 Table — Strains of C. neoformans and S. cerevisiae that are referenced in this work. The C. neoformans JEC21 strain was used to collect tRNA modification and transcriptome data reported here. (PDF) [file pone.0266239.s009.pdf]

S1 Table. Strains referenced and used in this study.

Strain	Data Type	Reference
C. neoformans JEC21	Transcriptome (IR)<br>tRNA modifications	This study
C. neoformans H99	Transcriptome (IR & H_2O_2)	29, 30
S. cerevisiae BY4741	tRNA modifications	17, 18, 19, 20
S. cerevisiae DBY7286	Transcriptome (H_2O_2)	31, 88
S. cerevisiae S288C	Codon usage	32, 34

Strains of *C. neoformans* and *S. cerevisiae* that are referenced. The *C. neoformans* JEC21 strain was used to collect tRNA modification and transcriptome data reported here.
