# Supplementary material for: Ionizing radiation and chemical oxidant exposure impacts on Cryptococcus neoformans transfer RNAs
Source: PLoS One. 2022 Mar 29;17(3):e0266239. doi: 10.1371/journal.pone.0266239 (PMC8963569; doi:10.1371/journal.pone.0266239)
Supplement: S2 Table — The primers for GAPDH, CAT1, and Trm7 were designed using Primer-BLAST [20]. (PDF) [file pone.0266239.s010.pdf]

S2 Table. Primers used for qPCR experiments in this study.

Gene	*C. neoformans* ID		Sequence 5' - 3'	Tm (°C)	Product Size (bp)
GAPDH	CNF03160	Forward<br>Reverse	GAC CTG CGC AAC AGA GTC AG<br>AAC GTT CAG GTA GGA ACG CC	58°C	189
Trm7	CNB02570	Forward<br>Reverse	GTA TGT GGG AAG CTG AGG GC<br>TCC GAC ATC CAG CAA CAT GG	58°C	138
CAT1	CNL06020	Forward<br>Reverse	TGA GGT GCT TGT CAG TCT AGG<br>AGA GAA TGG CAA CGC TCT GG	57°C	139

The primers for GAPDH, CAT1, and Trm7 were designed using Primer-BLAST [20].
