# Supplementary material for: Ionizing radiation and chemical oxidant exposure impacts on Cryptococcus neoformans transfer RNAs
Source: PLoS One. 2022 Mar 29;17(3):e0266239. doi: 10.1371/journal.pone.0266239 (PMC8963569; doi:10.1371/journal.pone.0266239)
Supplement: S3 Table — Genes reported to be upregulated in response to H2O2 in C. neoformans [29]. These genes were used for codon usage analyses in S3 and S4 Figs. (PDF) [file pone.0266239.s011.pdf]

S3 Table. List of the genes in *C. neoformans* H99 (and corresponding JEC21 strain gene) induced by H_2O_2 and IR exposure.

**Standard**	**JEC21 gene**	**Protein Function**
CAT1	CNL06020	Protects cell from toxic effects of H_2O_2
NDE2	CNG00750	Mitochondrial NADH dehydrogenase, putative
OLE1	CNJ01180	Stearoyl-CoA 9-desaturase
SCS7	CNK00680	Oxidoreductase, putative
SUR2	CNB03230	Sphingosine hydroxylase, putative
CYB25	CNN01090	Cytoplasm protein, putative
KGD1	CNB01730	Oxoglutarate dehydrogenase (succinyl-transferring), putative
BDH1	CNM01710	(R,R)-butanediol dehydrogenase, putative
RAD54	CND02890	DNA supercoiling, putative
RAD51	CNA06990	Recombinase
RDH54	CNC03920	DNA supercoiling
RFA1	CND02690	Damaged DNA binding protein
MRE11	CNE00700	Meiotic DNA double-strand break processing related protein
DNL4	CNK00930	DNA ligase (ATP)
RFA2	CND04360	Hypothetical protein
SOD1	CND01490	Copper zinc superoxide dismutase

Genes reported to be upregulated in response to H_2O_2 and IR in *C. neoformans* H99 strain [29, 30]. The corresponding gene was identified in the JEC21 strain and then used for codon usage analyses in S3 and S4 Figs.
