# Supplementary material for: Ionizing radiation and chemical oxidant exposure impacts on Cryptococcus neoformans transfer RNAs
Source: PLoS One. 2022 Mar 29;17(3):e0266239. doi: 10.1371/journal.pone.0266239 (PMC8963569; doi:10.1371/journal.pone.0266239)
Supplement: S4 Table — Genes in S. cerevisiae reported to be upregulated by H2O2 exposure [31]. These genes were used for codon usage analyses in S5 Fig. (PDF) [file pone.0266239.s012.pdf]

S4 Table. List of the genes in *S. cerevisiae* induced by H_2O_2 exposure.

Standard	Gene	Protein Function
TRX2	YGR209C	Cytoplasmic thioredoxin isoenzyme; protects cell against oxidative and reductive stress
TSA2	YDR453C	Stress inducible cytoplasmic thioredoxin peroxidase; removal of reactive oxygen
	YML131W	Cytoplasmic protein with unknown function
	YNL134C	NADH-dependent aldehyde reductase
GRE2	YOL151W	Stress-induced 3-methylbutanol reductase and NADPH-dependent methylglyoxal reductase
	YDL124W	NADPH-dependent alpha-keto amide reductase
TTR1/GRX2	YDR513W	Cytoplasmic glutaredoxin; thioltransferase
PRX1	YBL064C	Mitochondrial peroxiredoxin with thioredoxin peroxidase activity
	YMR090W	Putative cytoplasmic protein of unknown function

Genes in *S. cerevisiae* reported to be upregulated by H_2O_2 exposure [31]. These genes were used for codon usage analyses in S5 Fig.
