# Supplementary material for: Ionizing radiation and chemical oxidant exposure impacts on Cryptococcus neoformans transfer RNAs
Source: PLoS One. 2022 Mar 29;17(3):e0266239. doi: 10.1371/journal.pone.0266239 (PMC8963569; doi:10.1371/journal.pone.0266239)
Supplement: S5 Table — The most abundant proteins [34] were assumed to be representative of the most abundant transcripts and used for codon usage analyses in S3 and S4 Figs. (PDF) [file pone.0266239.s013.pdf]

S5 Table. List of the most abundant proteins in *C. neoformans*.

**Systematic**	**Protein Function**
CNC06180	Hypothetical protein
UBI1	Ubiquitin-carboxy extension protein function
CNA06950	Electron carrier; final protein carrier in electron-transport chain
CNI01590	Manganese superoxide dismutase; destroys radicals produced within the cell
TEF1	Translation elongation factor EF-α; promotes the binding of aminoacyl-tRNA to the A-site of ribosomes during protein synthesis
CNG04230	Hypothetical protein
CNK03170	Transaldolase; balances metabolites in pentose-phosphate pathway
CNE03920	60S ribosomal protein I4-a

The most abundant proteins [34] were assumed to be representative of the most abundant transcripts and used for codon usage analyses.
