# Supplementary material for: Ionizing radiation and chemical oxidant exposure impacts on Cryptococcus neoformans transfer RNAs
Source: PLoS One. 2022 Mar 29;17(3):e0266239. doi: 10.1371/journal.pone.0266239 (PMC8963569; doi:10.1371/journal.pone.0266239)
Supplement: S6 Table — The most abundant proteins [34] were assumed to be representative of the most abundant transcripts and used for codon usage analyses in S5 Fig. (PDF) [file pone.0266239.s014.pdf]

S6 Table. List of the most abundant proteins in *S. cerevisiae*.

Standard	Systematic	Protein Function
FBA1	YNK060C	Fructose 1,6-bisphosphate aldolase; required for glycolysis
ENO2	YHR174W	Enolase 2; catalyzes conversion during glycolysis
PGK1	YCR012W	3-phosphoglycerate kinase
CDC19	YAL038W	Pyruvate kinase
TDH3	YGR192C	Glyceraldehyde-3-phosphate dehydrogenase
PDC1	YLR044C	Major of 3 pyruvate decarboxylase isozymes
SSA2	YLL024C	Hsp70 family; ATP-binding protein involved in protein folding
PMA1	YGL008C	Plasma membrane proton-ATPase

The most abundant proteins [34] were assumed to be representative of the most abundant transcripts and used for codon usage analyses.
