# Supplementary material for: Ionizing radiation and chemical oxidant exposure impacts on Cryptococcus neoformans transfer RNAs
Source: PLoS One. 2022 Mar 29;17(3):e0266239. doi: 10.1371/journal.pone.0266239 (PMC8963569; doi:10.1371/journal.pone.0266239)
Supplement: S7 Table — Homologues of UUG-enriched genes in S. cerevisiae were identified in C. neoformans and used for codon usage analyses in Fig 4. (PDF) [file pone.0266239.s015.pdf]

S7 Table. List of homologous genes identified in *C. neoformans*.

Abundant Genes in *S. cerevisiae*	ID in *C. neoformans*	E-value	Protein Function in *C. neoformans*
FBA1	CNB00300	$2E^{-36}$	Fructose-bisphosphate aldolase, putative
ENO2	CNC00160	$3E^{-48}$	Phosphopyruvate hydratase, putative
PGK1	CNG02220	$8E^{-36}$	Phosphoglycerate kinase, putative
CDC19	CNC03080	$1E^{-90}$	Pyruvate kinase, putative
TDH3	CNF03160	$6E^{-11}$	Glyceraldehyde-3-phosphate dehydrogenase
PDC1	CNJ00950	$3E^{-05}$	Pyruvate decarboxylase, putative
SSA1	CNC02520	0	Chaperone, putative
PMA1	CNG00380	$3E^{-46}$	Hydrogen-exporting ATPase, putative

Homologues of UUG-enriched genes in *S. cerevisiae* were identified in *C. neoformans* and used for codon usage analyses in Fig 4.
